# Integrin αvβ6‐Targeted PET/CT Imaging of Non‐Small Cell Lung Cancer with [^68^Ga]Ga‐Trivehexin: Improved Preoperative Lymph Node Staging and Association with Immunohistochemistry

**DOI:** 10.1002/advs.202508225

**Published:** 2025-09-03

**Authors:** Huiqin Wu, Chongjiao Li, Ling Li, Yueli Tian, Zhiwei Xiao, Yiwei Huang, Juan Zhong, Jianying Huang, Qiongrong Chen, Yong He

**Affiliations:** ^1^ Department of Nuclear Medicine Zhongnan Hospital of Wuhan University No. 169 East Lake Road, Wuchang District Wuhan Hubei Province 430071 China; ^2^ Department of Pathology Zhongnan Hospital of Wuhan University Wuhan 430071 China; ^3^ Clinical Trial Center Zhongnan Hospital of Wuhan University Wuhan 430071 China

**Keywords:** non‐small cell lung cancer, integrin αvβ6, [^68^Ga]Ga‐Trivehexin, [^18^F]FDG, PET/CT

## Abstract

Integrin αvβ6, an epithelial‐specific integrin, plays a crucial role in the development and prognosis of multiple tumors and has emerged as a potential target for cancer diagnosis and treatment. The novel integrin αvβ6‐targeted radiotracer [^68^Ga]Ga‐Trivehexin shows excellent targeting specificity and favorable tumor‐to‐background contrast. In this prospective clinical study (NCT05835570) involving 58 participants with non‐small cell lung cancer (NSCLC), [^68^Ga]Ga‐Trivehexin and 2‐deoxy‐2‐[^18^F]fluoro‐*D*‐glucose ([^18^F]FDG) positron emission tomography/computed tomography (PET/CT) showed perfect diagnostic accuracy (100%, 58/58) for the detection of primary tumors. In detecting lymph node metastasis, [^68^Ga]Ga‐Trivehexin exhibited similar diagnostic sensitivity to [^18^F]FDG PET/CT (80.0% vs 72.0%), with higher specificity (93.8% vs 62.5%, *p *< 0.001) and accuracy (91.2% vs 64.2%, *p *< 0.001) in per‐station analysis. [^68^Ga]Ga‐Trivehexin outperformed [^18^F]FDG in detecting brain metastasis (sensitivity, 92.3% vs 38.5%; *p *= 0.031). Additionally, [^68^Ga]Ga‐Trivehexin PET/CT results changed the therapeutic regimens of 14 (24.1%) participants. The maximum standardized uptake value (SUVmax) and tumor‐to‐liver parenchyma ratio (TLR) derived from [^68^Ga]Ga‐Trivehexin correlated with integrin β6 expression in NSCLC. Overall, [^68^Ga]Ga‐Trivehexin PET/CT represents an invaluable tool for identifying lymph node and brain metastases and shows comparable diagnostic efficacy in detecting primary tumors and other distant metastases compared with [^18^F]FDG in NSCLC, potentially guiding treatment decision‐making.

## Graphical abstract

Integrin αvβ6‐targeted [^68^Ga]Ga‐Trivehexin positron emission tomography/computed tomography (PET/CT) can accurately identify regional lymph nodes and cerebral metastases in non‐small cell lung cancer (NSCLC) compared with 2‐deoxy‐2‐[^18^F]fluoro‐*D*‐glucose ([^18^F]FDG). [^68^Ga]Ga‐Trivehexin uptake positively correlates with integrin β6 expression in NSCLC. These findings highlight the great potential of [^68^Ga]Ga‐Trivehexin PET/CT as a powerful tool for the diagnosis of NSCLC, potentially guiding patient treatment strategies.

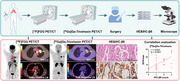

## 1 Introduction

Over the past two decades, encouraging advances have been made in the treatment of non‐small cell lung cancer (NSCLC), primarily from the development of targeted therapy and immunotherapy.^[^ ^1^, ^2^ ^]^ Significant survival benefits have been achieved from these treatments in patients with NSCLC, with the 3‐year survival rate increasing from 26% to 40% in the past 10 years. Nevertheless, many deaths are still caused by NSCLC each year, partly because the disease is usually in the advanced or late stage at the time of initial diagnosis. Early diagnosis of NSCLC remains challenging in part because of the limited diagnostic sensitivity of conventional imaging modalities.^[^ ^3^ ^]^ Positron emission tomography/computed tomography (PET/CT) using 2‐deoxy‐2‐[^18^F]fluoro‐*D*‐glucose ([^18^F]FDG), an imaging modality that combines both metabolic and anatomical information, plays an important role in the early diagnosis and clinical management of lung cancer.^[^ ^4^, ^5^, ^6^, ^7^ ^]^ However, infectious disease can lead to false‐positive findings in pulmonary lesions or mediastinal lymph nodes, which greatly restricts the diagnostic specificity of [^18^F]FDG.^[^ ^8^ ^]^ Therefore, there is an urgent need to identify robust biomarkers and develop novel imaging agents to facilitate more precise diagnosis and treatment of NSCLC.

Integrins are a class of transmembrane receptor proteins that function as heterodimers comprising two subunits, α and β. Thus far, 18 α and 8 β subunits have been identified. The integrin β6 subunit exclusively binds the αv subunit to form integrin αvβ6; therefore, integrin β6 is the crucial subunit in determining integrin αvβ6 levels and functions.^[^ ^9^ ^]^ Integrin αvβ6 specifically recognizes and binds to extracellular latency‐associated peptide, which contains arginine‐glycine‐aspartic (RGD) tripeptides, leading to the release of transforming growth factor β1 (TGFβ1), the key ligand that induces αvβ6 to promote cancer and fibrosis.^[^ ^10^, ^11^ ^]^ A growing body of literature has shown that integrin αvβ6 is closely related to tumor proliferation, migration, invasion, angiogenesis, and epithelial‐mesenchymal transition (EMT). Moreover, integrin αvβ6 is associated with poor clinical outcomes in multiple malignant tumors due to its roles in mediating the activation and release of TGFβ1.^[^ ^12^, ^13^, ^14^ ^]^ The expression of integrin αvβ6 is tumor‐specific and highly upregulated in multiple tumors, including lung cancer, whereas the expression of αvβ6 in normal tissues and organs is typically low or undetectable.^[^ ^15^, ^16^, ^17^, ^18^ ^]^ Therefore, the development of integrin αvβ6‐targeted diagnostic and therapeutic agents represents a promising direction for cancer research.

Numerous peptide‐based integrin αvβ6‐targeted PET imaging agents have been explored for detecting malignant tumors.^[^ ^19^, ^20^ ^]^ While some integrin αvβ6‐targeted PET probes, including [^18^F]‐αvβ6‐BP, [^68^Ga]Ga‐SFLAP3, and [^68^Ga]Ga‐DOTA‐SFITGv6, have been synthesized and translated into clinical trials, data from only a limited number of cancer patients and healthy volunteers have been published.^[^ ^21^, ^22^, ^23^, ^24^, ^25^, ^26^ ^]^ Furthermore, most of these probes have some limitations, including poor internal stability and prominent tracer uptake in the gastrointestinal tract. Recently, Notni and colleagues synthesized and optimized a novel cyclic peptide trimer probe targeting integrin αvβ6, named [^68^Ga]Ga‐Trivehexin, which exhibited higher target affinity (IC50 = 0.047 nM), enhanced tumor uptake, and prolonged retention time compared with another trimeric cyclic peptide probe [^68^Ga]Ga‐TRAP(AvB6).^[^ ^27^ ^]^ [^68^Ga]Ga‐Trivehexin shows favorable characteristics, including lower background uptake and improved tumor delineation. Aside from predominant renal excretion, only slight physiological uptake was observed in the gastrointestinal tract, allowing for clear detection of abdominal and pelvic lesions.^[^ ^27^ ^]^ [^68^Ga]Ga‐Trivehexin has exhibited great diagnostic potential for detecting malignancies, such as pancreatic cancer and head and neck malignant tumors.^[^ ^28^ ^]^ However, the diagnostic utility of [^68^Ga]Ga‐Trivehexin in the detection and staging of NSCLC has not been systematically investigated. We hypothesized that [^68^Ga]Ga‐Trivehexin holds substantial potential for identifying primary tumors and metastatic lesions in NSCLC. We therefore conducted a prospective study to evaluate the diagnostic efficiency of [^68^Ga]Ga‐Trivehexin PET/CT compared with [^18^F]FDG PET/CT. We further explored the intrinsic correlations between [^68^Ga]Ga‐Trivehexin uptake and integrin β6 expression in NSCLC.

## 2 Results

### 2.1 Participant Characteristics

**Figure 1:**
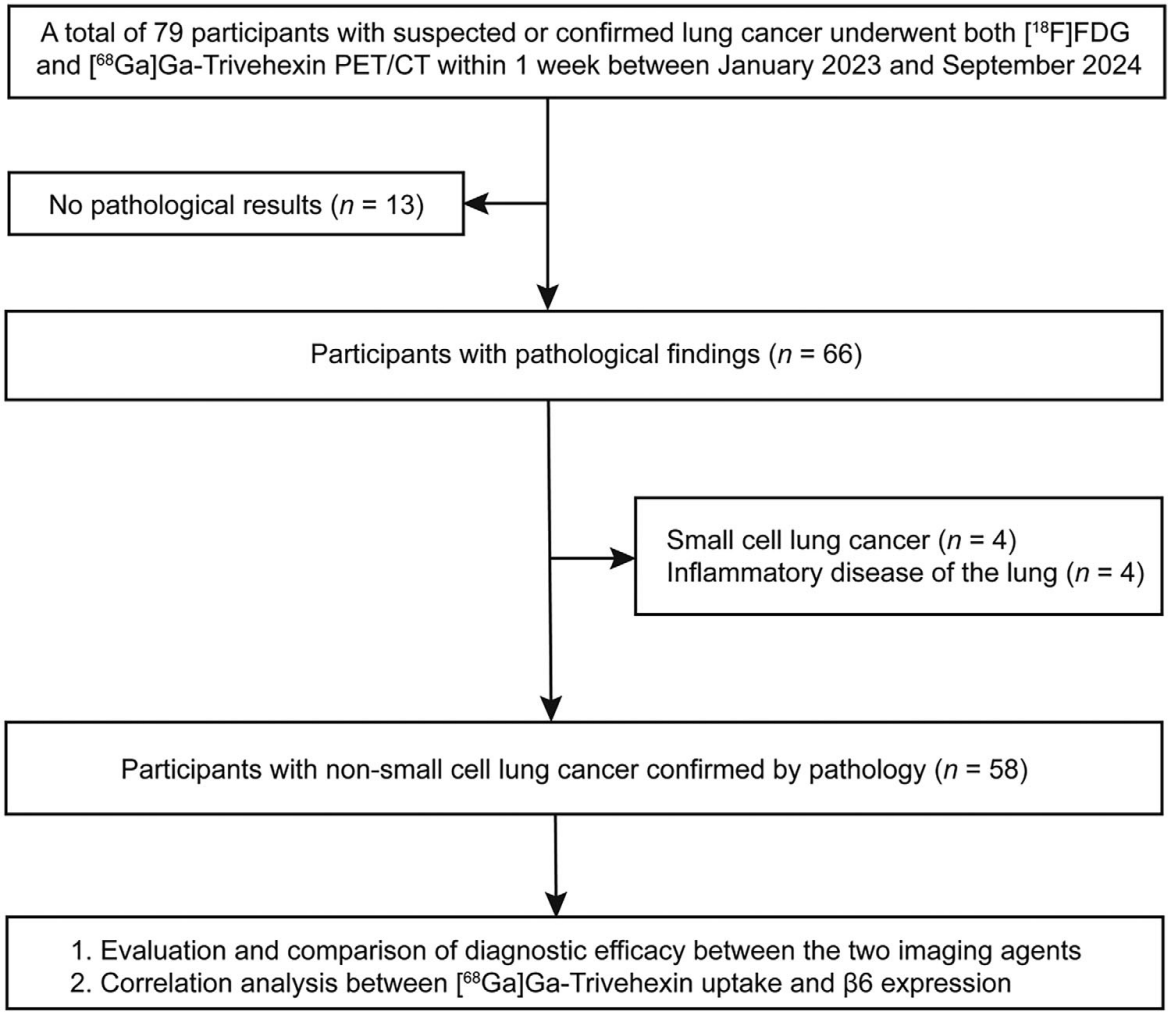
Flowchart for the participant selection process.

Seventy‐nine participants were initially enrolled in this study. From this group, 21 were excluded, including 13 without pathological information, 4 with small cell lung cancer (SCLC), and 4 with non‐neoplastic disease. Finally, 58 participants with pathologically confirmed NSCLC were analyzed. The detailed participant selection process is shown in **Figure** **1**.

The cohort of 58 participants (45 men; mean age, 62.6 ± 8.4 years) included 30 cases of adenocarcinoma, 19 cases of squamous cell carcinoma, four cases of large cell neuroendocrine carcinoma, two cases of pulmonary mucinous adenocarcinoma, and 1 case each of adenosquamous carcinoma, pulmonary sarcomatoid carcinoma, and a non‐special type of NSCLC. The detailed clinicopathological features of the study participants are presented in Table S1 (Supporting Information).

### 2.2 Primary Tumor Detection

**Figure 2:**
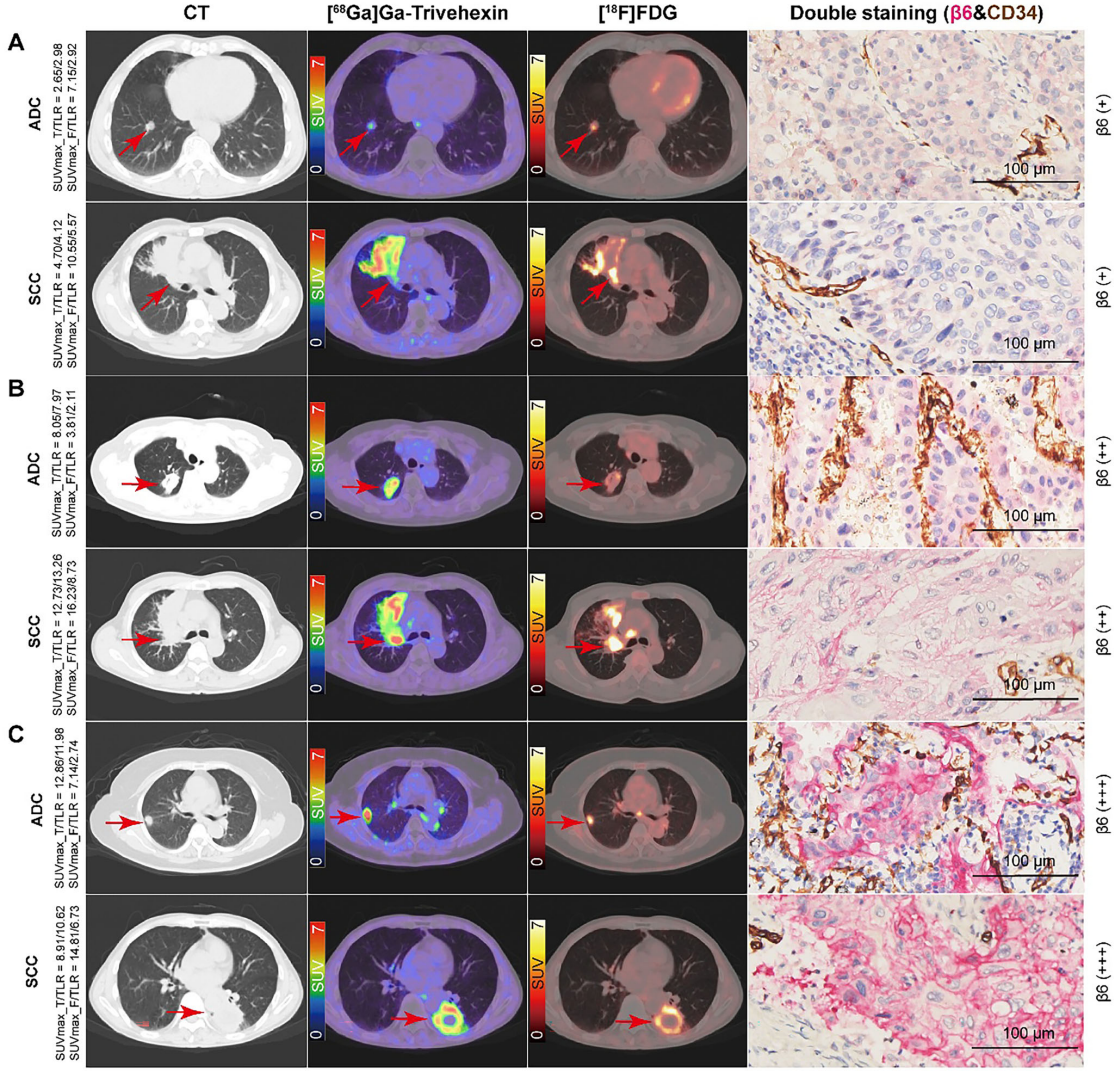
Representative chest CT, [^68^Ga]Ga‐Trivehexin, [^18^F]FDG PET/CT, and double immunostaining (integrin β6 and CD34) images of non‐small cell lung cancer cases. (A) Top row: A 61‐year‐old man with lung adenocarcinoma (ADC, arrows; pT1bN1M0, IIB) in the right upper lobe, exhibiting weak integrin β6 expression in the tumor. Bottom row: A 68‐year‐old man with squamous cell carcinoma (SCC, arrows; pT2aN0M0, IB) in the right upper lobe, showing weak integrin β6 expression in the tumor. (B) Top row: A 68‐year‐old woman with ADC (arrows; pT2aN0M0, IB) showing moderate integrin β6 expression in the tumor. Bottom row: A 58‐year‐old man with SCC (arrows; pT2aN2M0, IIIA) demonstrating moderate integrin β6 expression in the tumor. (C) Top row: A 69‐year‐old woman with ADC (arrows; pT1cN0M0, IA3) exhibiting strong integrin β6 expression in the tumor. Bottom row: A 64‐year‐old man with SCC (arrows; pT2bN0M0, IIA) showing a strong integrin β6 expression in the tumor. In double immunostaining, the rose‐red color indicates integrin β6 expression and the brown color indicates expression of CD34, which marks neovascularization within the tumor stroma. Magnification ×100; scale bar = 100 µm. SUVmax_T, [^68^Ga]Ga‐Trivehexin‐derived SUVmax; SUVmax_F, [^18^F]FDG‐derived SUVmax; TLR, tumor‐to‐liver parenchyma ratio.

A total of 58 primary tumors in the 58 treatment‐naïve participants were analyzed. All primary tumors were successfully detected on both [^68^Ga]Ga‐Trivehexin and [^18^F]FDG PET/CT, with an excellent detection rate of 100% (58/58) for both modalities. Representative cases are shown in **Figure** **2**. The median largest diameter of tumors was 3.65 cm. The median maximum standardized uptake value (SUVmax) of primary tumors on [^68^Ga]Ga‐Trivehexin was lower than that on [^18^F]FDG PET/CT (8.74 vs 15.12, *p* < 0.001), but the tumor‐to‐liver parenchyma ratio (TLR) of [^68^Ga]Ga‐Trivehexin was significantly higher than that of [^18^F]FDG (8.82 vs 5.98, *p* < 0.001) (**Table** **1**). On [^68^Ga]Ga‐Trivehexin PET/CT, larger tumors (> 3 cm) exhibited a distinctly higher median SUVmax (10.82 vs 5.67), TLR (9.79 vs 6.47), tumor‐to‐background (mediastinal blood pool) ratio (TBR) (9.45 vs 5.30), integrin β6 expression tumor volume (ITV) (24.37 vs 4.84 cm^3^), and total lesion integrin β6 expression (TLI) (132.96 vs 16.26) compared with smaller tumors (all *p* < 0.001). The same trends in the abovementioned indicators were observed with [^18^F]FDG PET/CT (all *p* < 0.05). The median [^18^F]FDG‐derived TBR and TLR of primary tumors were higher in participants with advanced NSCLC (stage III–IV) and non‐adenocarcinoma histology than those with stage I–II and adenocarcinoma. The median TBR and TLR of primary tumors on [^68^Ga]Ga‐Trivehexin PET/CT were not influenced by TNM stage or pathology type (Table S2, Supporting Information).

**Table 1:** Comparison of tracer uptake in primary tumors and metastatic lesions between [**^68^** Ga]Ga‐Trivehexin and [**^18^** F]FDG in non‐small **c**ell lung cancer.

Location of Lesions	[^68^Ga]Ga‐Trivehexin	[^18^F]FDG	*p* Value
	Median (IQR)	Median (IQR)	
**Primary tumor**			
No. of Lesions	58	58	
SUVmax	8.74 (5.78, 12.68)	15.12 (11.00, 18.86)	**< 0.001**
TBR	8.07 (5.36, 10.38)	7.91 (4.85, 11.19)	0.858
TLR	8.82 (6.10, 11.44)	5.98 (4.85, 8.57)	**< 0.001**
**Pleural metastasis** ^a)^			
No. of Lesions	49	49	
SUVmax	5.85 (4.01, 13.39)	19.25 (5.58, 30.22)	**< 0.001**
TBR	4.23 (2.97, 9.69)	12.22 (3.54, 19.19)	**0.003**
TLR	5.63 (3.80, 12.89)	8.38 (2.49, 13.15)	0.969
**Bone metastasis**			
No. of Lesions	41	45	
SUVmax	8.96 (6.03, 14.17)	10.34 (7.15, 14.94)	0.221
TBR	8.25 (5.49, 12.37)	5.74 (4.38, 9.41)	**0.035**
TLR	10.73 (7.32, 15.37)	4.48 (3.42, 7.23)	< 0.001
**Brain metastasis**			
No. of Lesions	12	5	
SUVmax	4.45 (3.11, 6.80)	9.44 (7.50, 11.33)	**0.004**
TBR_b_	177.36 (73.75, 212.70)	1.08 (1.04, 1.36)	**< 0.001**
TLR	4.59 (1.57, 9.19)	3.76 (2.05, 4.52)	0.721
**Liver metastasis**			
No. of Lesions	7	8	
SUVmax	10.58 (9.50, 22.72)	13.60 (6.33, 22.40)	0.728
TBR	8.30 (7.46, 20.96)	8.84 (2.73, 15.04)	0.355
TLR	12.42 (11.15, 36.01)	6.56 (2.18, 11.00)	**0.021**
**Other distant metastasis** ^b)^			
No. of Lesions	28	27	
SUVmax	4.67 (2.32, 6.09)	7.73 (3.60, 12.56)	**0.004**
TBR	3.66 (1.98, 4.39)	5.19 (2.70, 8.43)	**0.025**
TLR	3.49 (2.46, 5.72)	3.80 (1.92, 6.17)	0.987

Note. TBR, tumor‐to‐background (mediastinal blood pool) ratio; TLR, tumor‐to‐liver parenchyma ratio; TBR_b_, tumor‐to‐background (normal brain parenchyma) ratio. *p* < 0.05 represents a statistical difference.

^a)^ The location of pleural metastasis includes the pleura and the pericardium.

^b)^ Other distant metastatic locations include abdominal lymph node, lung, muscle, pancreas, peritoneum, and adrenal gland.

### 2.3 Evaluation of Lymph Node Metastasis

**Figure 3:**
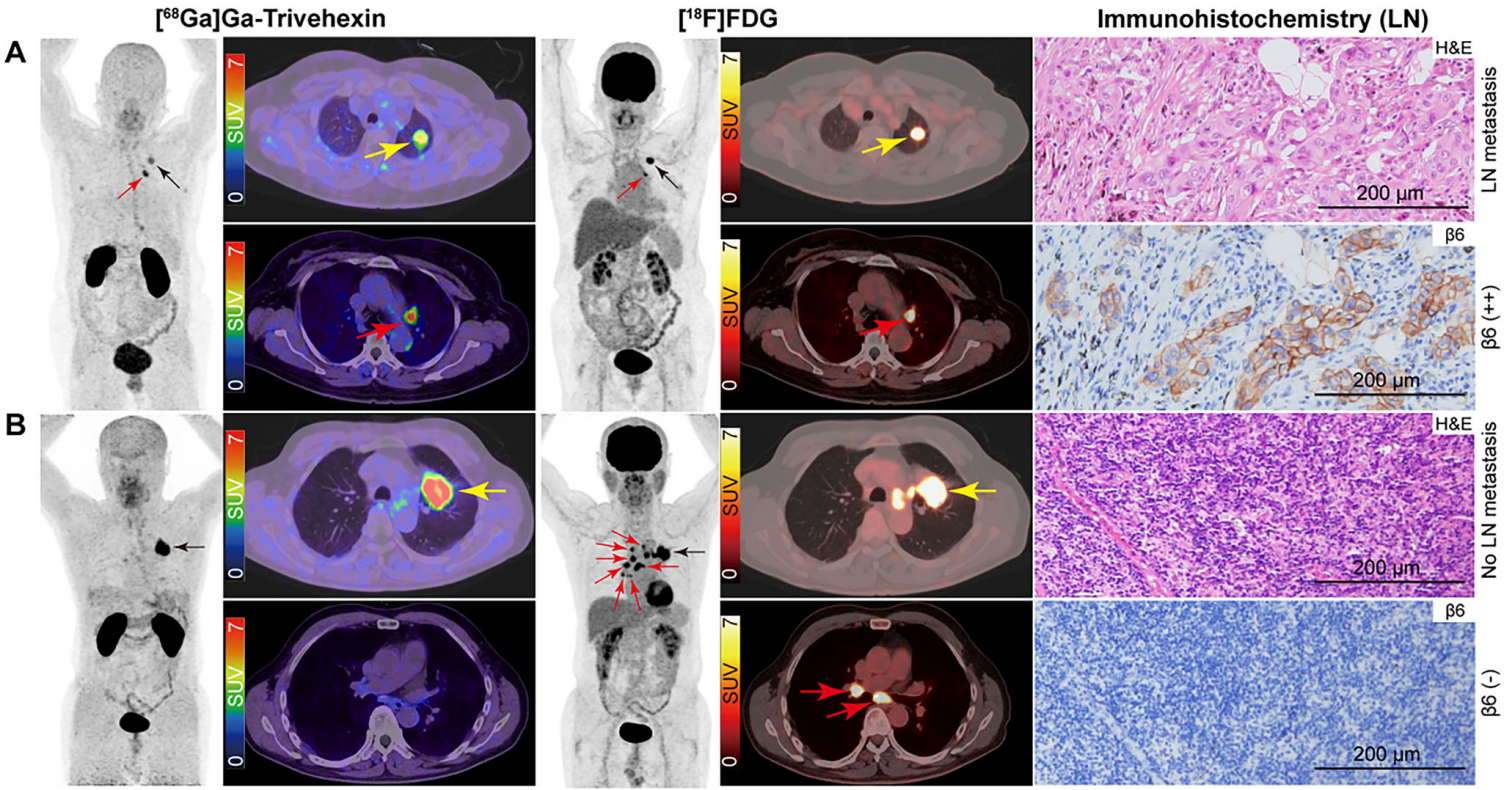
Representative true‐positive (A) and true‐negative (B) lymph nodes on [^68^Ga]Ga‐Trivehexin and corresponding hematoxylin‐eosin (H&E) and integrin β6 immunostaining. (A) A 54‐year‐old woman with left lung adenocarcinoma (ADC; black and yellow arrows) with ipsilateral mediastinal lymph nodes metastases (pT1cN2M0, IIB; red arrows). The [^68^Ga]Ga‐Trivehexin‐ and [^18^F]FDG‐derived SUVmax values were 4.92 and 12.58, respectively, for the primary tumor, and 8.48 and 7.10, respectively, for lymph nodes. H&E revealed metastasis to the lymph node, and immunohistochemistry of the metastatic lymph node showed moderate integrin β6 expression. (B) A 68‐year‐old woman with left lung adenocarcinoma (ADC; pT2aN0M0, IB; black and yellow arrows). [^18^F]FDG revealed multiple false‐positive lymph nodes in the mediastinum and bilateral hilum (red arrows), but these lymph nodes were true‐negative on [^68^Ga]Ga‐Trivehexin PET/CT. Histopathological findings revealed no lymph node metastasis and negative integrin β6 expression. Magnification ×100; scale bar = 200 µm.

In the overall cohort, 23 participants underwent pulmonary surgery with lymph node dissection, yielding pathology results for 337 lymph nodes, including 23 metastatic lymph nodes. Additionally, 16 regional lymph nodes were confirmed to have metastases by needle biopsy. In total, 353 lymph nodes located in 137 stations had pathological confirmation, including 39 metastatic and 314 non‐metastatic lesions. In the per‐station analysis, the sensitivity of [^68^Ga]Ga‐Trivehexin for detecting lymph node metastases tended to be higher than that of [^18^F]FDG (80.0% [20/25] vs 72.0% [18/25]), but without statistical significance (*p* = 0.500). However, [^68^Ga]Ga‐Trivehexin exhibited significantly higher specificity (93.8% [105/112] vs 62.5% [70/112], *p* < 0.001) and accuracy (91.2% [125/137] vs 64.2% [88/137], *p* < 0.001) compared with [^18^F]FDG PET/CT (**Table** **2**). In the per‐lesion analysis, the sensitivity, specificity, and accuracy were 61.5% (24/39), 97.1% (305/314), and 93.2% (329/353) for [^68^Ga]Ga‐Trivehexin and 56.4% (22/39), 80.6% (253/314) and 77.9% (275/353) for [^18^F]FDG, respectively (Table S3, Supporting Information). Representative cases are shown in **Figure** **3** and Figure S1 (Supporting Information).

**Table 2:** Diagnostic performance of [^68^Ga]Ga‐Trivehexin and [^18^F]FDG PET/CT for the detection of lymph node (LN) metastasis in non‐small cell lung cancer (station‐based analysis).

Location of LN	TP	FP	FN	TN	Sensitivity [%]	Specificity [%]	PPV [%]	NPV [%]	Accuracy [%]
**[^68^Ga]Ga‐Trivehexin PET/CT**									
**Station (2L)**	2	0	0	0	100 (2/2)	NA	100 (2/2)	NA	100 (2/2)
**Station (2R)**	1	0	0	5	100 (1/1)	100 (5/5)	100 (1/1)	100 (5/5)	100 (6/6)
**Station (3)**	0	0	0	1	NA	100 (1/1)	NA	100 (1/1)	100 (1/1)
**Station (4L)**	0	0	0	3	NA	100 (3/3)	NA	100 (3/3)	100 (3/3)
**Station (4R)**	5	2	0	9	100 (5/5)	81.8 (9/11)	71.4 (5/7)	100 (9/9)	87.5 (14/16)
**Station (5)**	1	1	1	10	50.0 (1/2)	90.9 (10/11)	50.0 (1/2)	90.9 (10/11)	84.6 (11/13)
**Station (6)**	1	0	0	1	100 (1/1)	100 (1/1)	100 (1/1)	100 (1/1)	100 (2/2)
**Station (7)**	3	1	1	21	75.0 (3/4)	95.5 (21/22)	75.0 (3/4)	95.5 (21/22)	92.3 (24/26)
**Station (8)**	0	0	0	1	NA	100 (1/1)	NA	100 (1/1)	100 (1/1)
**Station (9L)**	0	0	0	9	NA	100 (9/9)	NA	100 (9/9)	100 (9/9)
**Station (9R)**	0	0	0	6	NA	100 (6/6)	NA	100 (6/6)	100 (6/6)
**Station (10L)**	3	2	0	9	100 (3/3)	81.3 (9/11)	60.0 (3/5)	100 (9/9)	85.7 (12/14)
**Station (10R)**	1	0	1	6	50.0 (1/2)	100 (6/6)	100 (1/1)	85.7 (6/7)	87.5 (7/8)
**Station (11–12L)**	2	1	1	17	66.7 (2/3)	94.4 (17/18)	66.7 (2/3)	94.4 (17/18)	90.5 (19/21)
**Station (11–12R)**	1	0	1	7	50.0 (1/2)	100 (7/7)	100 (1/1)	87.5 (7/8)	88.9 (8/9)
**Total**	20	7	5	105	80.0 (20/25)	93.8 (105/112)	74.1 (20/27)	95.5 (105/110)	91.2 (125/137)
**[^18^F]FDG PET/CT**									
**Station (2L)**	2	0	0	0	100 (2/2)	NA	100 (2/2)	NA	100 (2/2)
**Station (2R)**	0	2	1	3	NA	60.0 (3/5)	NA	75.0 (3/4)	50.0 (3/6)
**Station (3)**	0	0	0	1	NA	100 (1/1)	NA	100 (1/1)	100 (1/1)
**Station (4L)**	0	2	0	1	NA	33.3 (1/3)	NA	100 (1/1)	33.3 (1/3)
**Station (4R)**	5	8	0	3	100 (5/5)	27.3 (3/11)	38.5 (5/13)	100 (3/3)	50.0 (8/16)
**Station (5)**	1	3	1	8	50.0 (1/2)	72.7 (8/11)	25.0 (1/4)	88.9 (8/9)	69.2 (9/13)
**Station (6)**	1	1	0	0	100 (1/1)	NA	50.0 (1/2)	NA	50.0 (1/2)
**Station (7)**	3	11	1	11	75.0 (3/4)	50.0 (11/22)	21.4 (3/14)	91.7 (11/12)	53.9 (14/26)
**Station (8)**	0	0	0	1	NA	100 (1/1)	NA	100 (1/1)	100 (1/1)
**Station (9L)**	0	0	0	9	NA	100 (9/9)	NA	100 (9/9)	100 (9/9)
**Station (9R)**	0	1	0	5	NA	83.3 (5/6)	NA	100 (5/5)	83.3 (5/6)
**Station (10L)**	3	6	0	5	100 (3/3)	45.5 (5/11)	33.3 (3/9)	100 (5/5)	57.1 (8/14)
**Station (10R)**	1	5	1	1	50.0 (1/2)	16.7 (1/6)	16.7 (1/6)	50.0 (1/2)	25.0 (2/8)
**Station (11–12L)**	1	2	2	16	33.3 (1/3)	88.9 (16/18)	33.3 (1/3)	88.9 (16/18)	81.0 (17/21)
**Station (11–12R)**	1	1	1	6	50.0 (1/2)	85.7 (6/7)	50.0 (1/2)	85.7 (6/7)	77.8 (7/9)
**Total**	18	42	7	70	72.0 (18/25)	62.5 (70/112)	30.0 (18/60)	90.9 (70/77)	64.2 (88/137)

Note. TP, true‐positive; FP, false‐positive; FN, false‐negative; TN, true‐negative; PPV, positive predictive value; NPV, negative predictive value; NA, not applicable

The median short diameter of metastatic lymph nodes was significantly larger than that of non‐metastatic lymph nodes (1.16 vs 0.66 cm, *p* < 0.001). With [^68^Ga]Ga‐Trivehexin, the median SUVmax (8.52 vs 1.10), TBR (7.17 vs 1.08), and TLR (7.69 vs 1.22) of metastatic lymph nodes were significantly higher than those of non‐metastatic lymph nodes in per‐station analysis (all *p* < 0.001). Similar findings were observed with [^18^F]FDG PET/CT (SUVmax, 9.09 vs 2.95; TBR, 5.39 vs 1.37; TLR, 4.15 vs 1.14; all *p* < 0.001) (Table S4, Supporting Information). The SUVmax from [^68^Ga]Ga‐Trivehexin was lower than that of [^18^F]FDG (8.52 vs 9.09, *p* = 0.006) in metastatic lymph nodes, but [^68^Ga]Ga‐Trivehexin showed a higher TLR (7.69 vs 4.15, *p* = 0.001). Receiver operating characteristic curve (ROC) analysis demonstrated that [^68^Ga]Ga‐Trivehexin had better detection efficiency for regional lymph node metastasis compared with [^18^F]FDG, with area under the curve (AUC) values of 0.980 and 0.928 for SUVmax, respectively, in a per‐station analysis (*p* = 0.0254) (Table S5 and Figure S2A, Supporting Information), and AUC values of 0.963 and 0.879 for SUVmax, respectively, in a per‐lesion analysis (*p* = 0.0106) (Table S5 and Figure S2B, Supporting Information). When an SUVmax of 2.35 was used as a threshold for [^68^Ga]Ga‐Trivehexin in per‐station analysis, the sensitivity, specificity, and accuracy for distinguishing lymph node metastasis were 95.5% (21/22), 96.8% (92/95), and 96.6% (113/117), respectively.

### 2.4 Detection of Distant Metastasis

**Figure 4:**
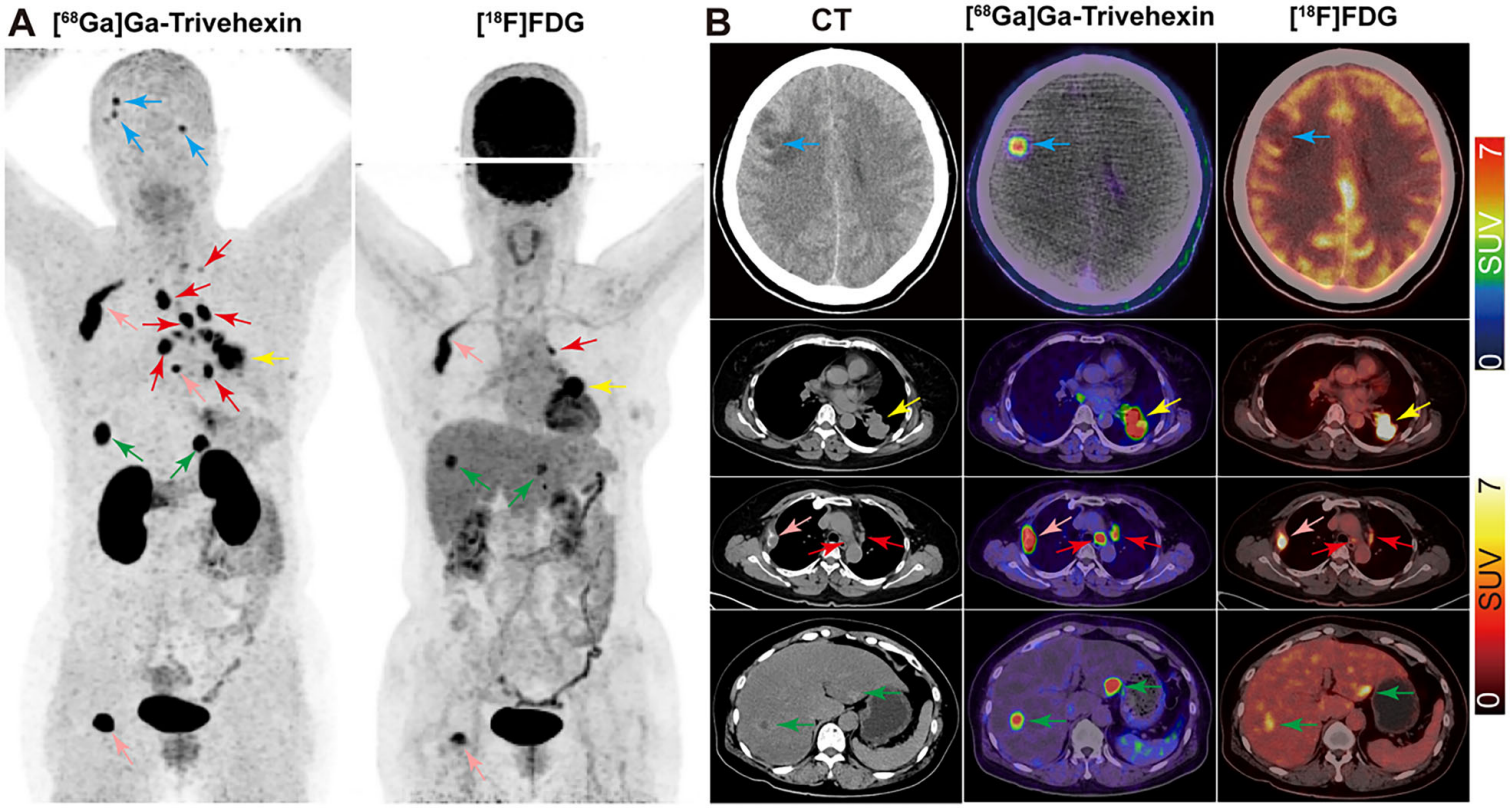
Representative [^68^Ga]Ga‐Trivehexin PET/CT images of a 55‐year‐old woman with lung adenocarcinoma and multiple metastatic lesions (cT2bN3M1c, IVB) compared with [^18^F]FDG PET/CT. The maximum intensity projection (MIP; A), axial CT, and fused images (B) of [^68^Ga]Ga‐Trivehexin and [^18^F]FDG PET/CT showed the primary tumor (SUVmax, 20.66 vs 12.45; yellow arrows) and metastatic lesions including lymph nodes (SUVmax, 23.88 vs 7.44; red arrows), brain (SUVmax, 3.39– 6.85 vs 9.78–12.89; TBR_b_, 96.23–195.66 vs 1.05–1.39; blue arrows), liver (SUVmax, 22.72 vs 7.05; green arrows) and bone metastases (SUVmax, 20.38 vs 11.19; pink arrows). TBR_b_, tumor‐to‐background (normal brain parenchyma) ratio.

Distant metastases occurred in 19 of the 58 participants and were detected in the pleura and pericardium, bone, brain, abdominal lymph node, liver, muscle, lung, pancreas, adrenal gland, and peritoneum. Compared with [^18^F]FDG, [^68^Ga]Ga‐Trivehexin exhibited great advantages in the detection of brain metastases because of its low background uptake in the normal brain parenchyma, with a per‐lesion sensitivity of 92.3% (12/13) for [^68^Ga]Ga‐Trivehexin compared with 38.5% (5/13) for [^18^F]FDG (*p* = 0.031). Representative cases are shown in **Figure** **4** and Figure S3A (Supporting Information). In the detection of other distant metastases, the two imaging agents showed similar diagnostic sensitivity (all *p* > 0.05) (Table S6, Supporting Information). Despite the lower median SUVmax of [^68^Ga]Ga‐Trivehexin in brain metastases compared with [^18^F]FDG (4.45 vs 9.44), a much higher median tumor‐to‐background (normal brain parenchyma) ratio (TBR_b_) was observed on [^68^Ga]Ga‐Trivehexin than [^18^F]FDG PET/CT (177.36 vs 1.08, respectively; *p* < 0.001). [^68^Ga]Ga‐Trivehexin showed a similar SUVmax compared with [^18^F]FDG in liver and bone metastases, but a higher TLR (Table 1).

### 2.5 Changes in T‐, N‐, and M‐staging and Therapeutic Management

**Figure 5:**
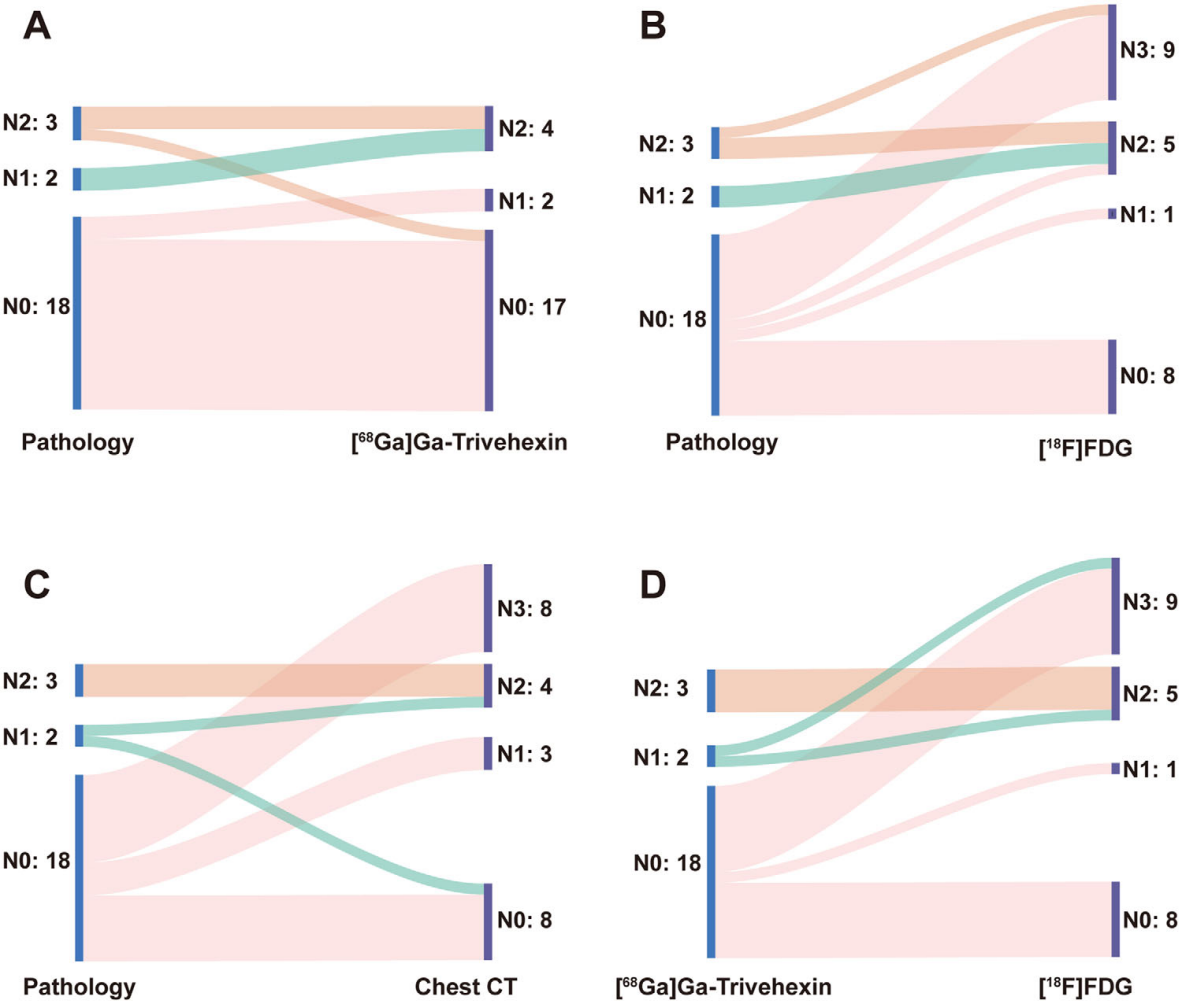
Sankey plots illustrating the changes in N‐staging. [^68^Ga]Ga‐Trivehexin (A), [^18^F]FDG (B), and chest CT (C) compared with surgical pathology and comparison between [^68^Ga]Ga‐Trivehexin and [^18^F]FDG (D) in non‐small cell lung cancer.

For N‐staging evaluation, 23 participants underwent mediastinal and hilar lymph node dissection, with pathology revealing 18 cases as N0, 2 as N1, and 3 as N2. [^68^Ga]Ga‐Trivehexin accurately predicted N‐staging in 18 participants (78.3%, 18/23), overestimated staging in 4 (17.4%, 4/23), and underestimated staging in 1 (4.4%, 1/23) (**Figure** **5**A). In contrast, [^18^F]FDG accurately predicted N‐staging in only 10 participants (43.5%, 10/23) and overestimated staging in 13 (56.5%, 13/23) (Figure 5B). Chest contrast‐enhanced CT accurately determined N‐staging in 10 participants (43.5%, 10/23), overestimated staging in 12 (52.2%, 12/23), and underestimated staging in 1 (4.4%, 1/23) (Figure 5C). Compared with [^18^F]FDG, [^68^Ga]Ga‐Trivehexin exhibited significantly higher diagnostic accuracy (78.3% vs 43.5%, *p* = 0.033) (Figure 5D). Of the 23 participants who underwent surgery, 12 (52.2%) eventually changed treatment regimens because of the superior specificity of [^68^Ga]Ga‐Trivehexin in detecting lymph node metastasis compared with [^18^F]FDG PET/CT, leading to N‐downstaging of participants.

For T‐ and M‐staging, both [^68^Ga]Ga‐Trivehexin and [^18^F]FDG showed an identical diagnostic accuracy of 100% in per‐patient analysis. Regarding clinical TNM stage, the diagnostic accuracy was 86.2% (50/58) with [^68^Ga]Ga‐Trivehexin and only 63.8% (37/58) with [^18^F]FDG PET/CT (*p* = 0.003). Treatment regimens were finally changed in 14 participants (24.1%, 14/58). Of these 14 participants, 12 were converted from the intended combination therapy to surgery alone or surgery with adjuvant chemotherapy, one was changed to stereotactic body radiation therapy, and one was redirected to radical concurrent chemoradiotherapy (Table S7, Supporting Information).

### 2.6 Assessment of the Correlation Between Tumor Tracer Uptake with Immunohistochemistry

**Figure 6:**
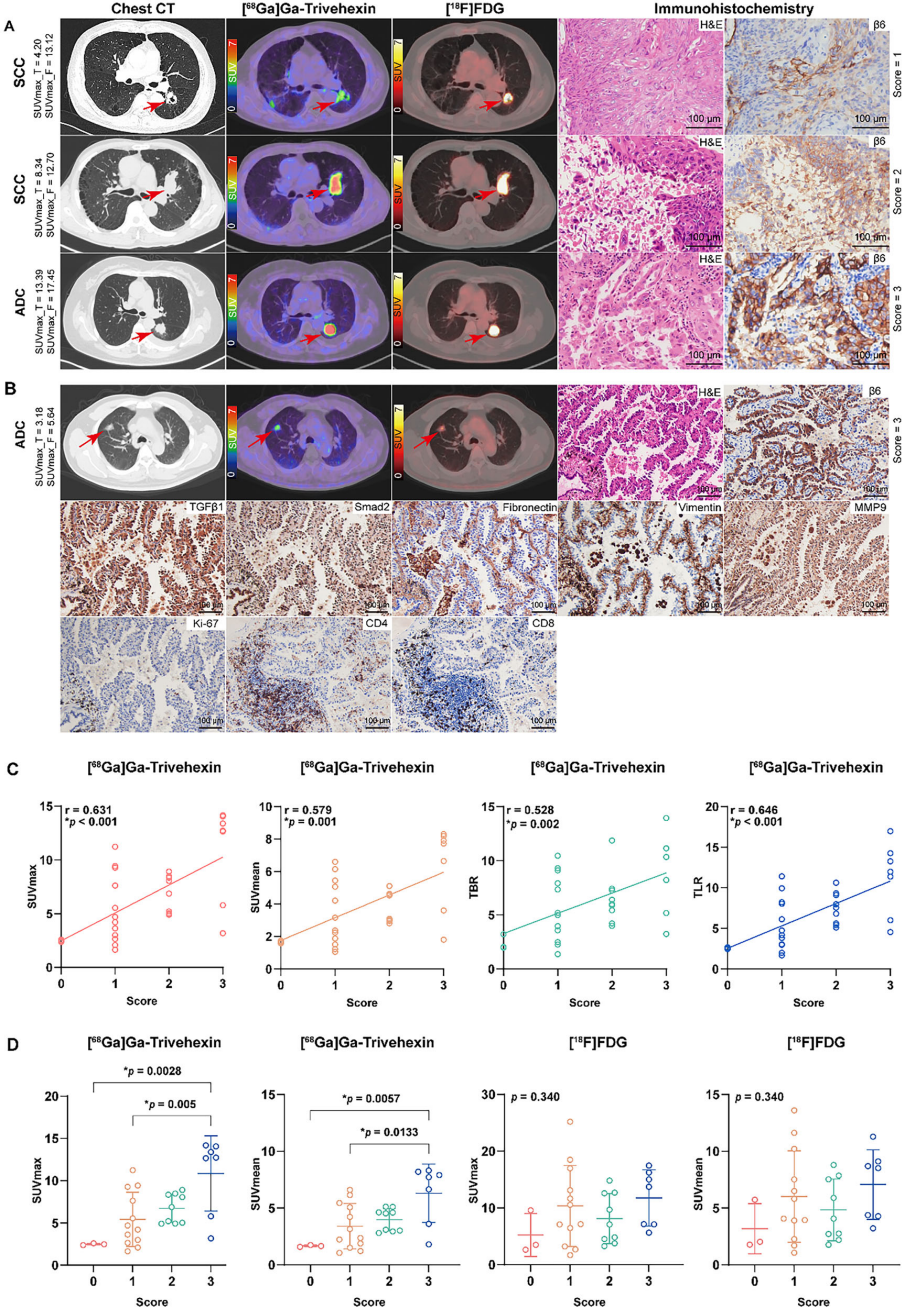
Representative chest CT, [^68^Ga]Ga‐Trivehexin, [^18^F]FDG PET/CT, tumor hematoxylin‐eosin (H&E) staining, and immunohistochemical staining images of non‐small cell lung cancer, correlation evaluation, and comparison of tumor tracer uptake between different integrin β6 score groups. (A) Top row: A 64‐year‐old man with lung squamous cell carcinoma (SCC; pT1cN0M0, IA3) in the inferior lobe of the left lung (arrows). Middle row: A 62‐year‐old man with lung SCC (pT1cN0M0, IA3) in the superior lobe of the left lung (arrows). Bottom row: A 66‐year‐old woman with lung adenocarcinoma (ADC; pT1cN0M0, IA3) in the inferior lobe of the left lung (arrows). (B) A 53‐year‐old man with ADC (pT1bN0M0, IA2) in the right upper lobe (arrows). Representative tumor H&E and immunohistochemical staining indicators (integrin β6, TGFβ1, Smad2, fibronectin, vimentin, MMP9, Ki‐67, CD4, and CD8) are shown. (C) Correlation analyses between [^68^Ga]Ga‐Trivehexin‐derived parameters (SUVmax, SUVmean, TBR, and TLR) and tumor tissue integrin β6 expression. (D) Comparison of tumor uptake (SUVmax and SUVmean) on [^68^Ga]Ga‐Trivehexin and [^18^F]FDG PET/CT between different integrin β6 score groups. Magnification ×100; scale bar = 100 µm. P‐values were determined by Spearman correlation coefficient (C), Kruskal‐Wallis *H* test, and Mann‐Whitney *U* test (D). *p < 0.05. SUVmax_T, [^68^Ga]Ga‐Trivehexin‐derived SUVmax; SUVmax_F, [^18^F]FDG‐derived SUVmax; TBR, tumor‐to‐background (mediastinal blood pool) ratio; TLR, tumor‐to‐liver parenchyma ratio.

Hematoxylin‐eosin (H&E), integrin β6, and TGFβ1 immunostaining were performed on 31 surgical tissue specimens from 23 primary tumors and 8 lymph nodes. Double staining for integrin β6 and CD34 confirmed that integrin β6 was expressed on the membrane of cancer cells but not in the neovascular endothelium within the tumor stroma (Figure 2). All primary cancer cells expressed integrin β6 with varying proportions, with especially stronger expression in the peripheric cancer cells adjacent to the tumor stroma (**Figure** **6**A; Figures S4 and S5, Supporting Information). Integrin β6 expression was observed in metastatic cancer cells in lymph nodes, but absent in non‐metastatic lymph nodes and normal lung tissues (Figure 3). In some cases, integrin β6 expression was also observed in the reactive hyperplastic alveolar epithelium that wrapped into the tumor stroma.

In this study, several protein molecules potentially related to integrin β6 were also detected. Immunohistochemistry results showed that TGFβ1 and SMAD family member 2 (Smad2) were expressed both in cancer cells and stromal cells, including lymphocytes, plasma cells, macrophages, and fibroblasts, in the tumor microenvironment. Fibronectin and vimentin were mainly expressed in the stromal cells and also detected in cancer cells in some cases of adenocarcinoma. Fibronectin and vimentin showed strong expression, with an IS score of 3, in almost all of the 10 examined tumor samples. Matrix metalloproteinase‐9 (MMP9) showed mild‐to‐intense expression in the cancer cells and stromal cells in the tumor microenvironment. The percentage of Ki‐67 varied widely among samples, from 1% to 70%. The number of CD8^+^ T cells was markedly more than that of CD4^+^ T cells in 60% (6/10) tumor samples, with a ratio of CD4/CD8 from 1:2 to 1:6. Representative immunohistochemistry images of primary tumors are shown in Figure 6B and Figure S6 (Supporting Information).

[^68^Ga]Ga‐Trivehexin‐derived SUVmax, mean SUV (SUVmean), TLI, TBR, and TLR showed moderate positive correlations with tumor integrin β6 expression (*r* = 0.631, 0.579, 0.460, 0.528, and 0.646, respectively, all *p* < 0.01) and TGFβ1 expression (*r* = 0.587, 0.593, 0.414, 0.551, and 0.553, respectively, all *p* < 0.03) (Figure 6C; Figure S7 and Table S8, Supporting Information) in NSCLC. Notably, we found that [^68^Ga]Ga‐Trivehexin‐derived SUVmax correlated with integrin β6 expression in pulmonary adenocarcinoma (*r* = 0.670, *p *= 0.003), but not in lung squamous cell carcinoma (*r* = −0.066, *p *= 0.857). The correlation was found between total lesion glycolysis (TLG) derived from [^18^F]FDG and integrin β6 expression (*r* = 0.373, *p* = 0.039) and TGFβ1 expression (*r* = 0.416, *p* = 0.020). [^68^Ga]Ga‐Trivehexin‐derived ITV and TLI were associated with the percentage of Ki‐67 in NSCLC (*r* = 0.739 and 0.652, *p* = 0.015 and 0.041). Similarly, [^18^F]FDG‐derived SUVmax, SUVmean, MTV, TLG, TBR, and TLR had significant associations with the percentage of Ki‐67 (*r* = 0.757, 0.702, 0.665, 0.708, 0.702, 0.739, respectively, all *p* < 0.05). [^68^Ga]Ga‐Trivehexin‐derived SUVmean and TLR and Smad2 showed a negative correlation trend (both *r* = −0.622, both *p *= 0.055) (Table S8, Supporting Information). No correlation was found between [^68^Ga]Ga‐Trivehexin uptake parameters and fibronectin, vimentin, or MMP9. Similarly, no association was observed between [^18^F]FDG‐derived parameters and Smad2, fibronectin, vimentin, or MMP9.

On [^68^Ga]Ga‐Trivehexin PET/CT, the median SUV of the lesions in the group with an immunohistochemistry score 3 for integrin β6 was significantly higher than that of the group with score 0 (12.73 vs 2.48 for SUVmax, *p* = 0.0028; 7.72 vs 1.58 for SUVmean, *p* = 0.0057) and score 1 (12.73 vs 4.70 for SUVmax, *p* = 0.005; 7.72 vs 3.16 for SUVmean, *p* = 0.0133). There was no significant difference in SUV on [^18^F]FDG PET/CT between the score groups (Figure 6D).

### 2.7 Assessment of the Relationship Between Tumor Tracer Uptake and Clinicopathological Features

Among the 23 participants who underwent surgery, five had well‐differentiated tumors, 11 had moderately differentiated tumors, and seven had poorly differentiated tumors. The SUVmax, SUVmean, TBR, and TLR derived from [^68^Ga]Ga‐Trivehexin showed moderately positive correlations with tumor histological grading in NSCLC, with correlation coefficients (*r*) of 0.489, 0.479, 0.475, and 0.597 (all *p* < 0.05), respectively. Similarly, [^18^F]FDG‐derived SUVmax also correlated with tumor histological grading (*r *= 0.425, *p* = 0.043), while SUVmean, TBR, and TLR showed no correlation with tumor histological grading (all *p* > 0.05) (Figure S8, Supporting Information). For all 58 participants, primary tumor uptake on [^68^Ga]Ga‐Trivehexin and [^18^F]FDG had a weak association with clinical TNM staging in NSCLC (all *p* < 0.05) (Figure S9, Supporting Information).

## 3 Discussion

Integrin αvβ6 is a heterodimeric transmembrane and epithelial‐specific receptor that is mainly regulated by the levels of the integrin β6 subunit. It is upregulated in various cancers and has emerged as an attractive molecular target for both diagnosis and therapy. The novel integrin αvβ6‐targeted tracer, [^68^Ga]Ga‐Trivehexin, was shown to exhibit excellent stability, exceptional affinity and selectivity, and promising imaging potential. In this study, we systematically investigated the clinical utility of [^68^Ga]Ga‐Trivehexin PET/CT for detecting primary and metastatic lesions in NSCLC and compared its diagnostic efficiency with [^18^F]FDG. Our results demonstrated that [^68^Ga]Ga‐Trivehexin PET/CT had higher specificity and accuracy in evaluating lymph node metastases compared with [^18^F]FDG, and its uptake positively correlated with integrin β6 expression in NSCLC.

The accurate detection of lung lesions is critical for treatment selection and prognosis evaluation. While integrin αvβ6‐targeted tracers have been comprehensively explored in preclinical studies, few imaging agents have been translated into clinical use for NSCLC. Flechsig et al. synthesized a probe, [^68^Ga]Ga‐SFITGv6, with high affinity (IC50 = 3.1 nM) and specificity demonstrated in vitro and in an in vivo xenograft model. However, in a clinical trial involving nine patients with NSCLC, [^18^F]FDG significantly outperformed [^68^Ga]Ga‐SFITGv6 in diagnostic accuracy.^[^ ^23^ ^]^ In our study, [^68^Ga]Ga‐Trivehexin exhibited an excellent detection rate of 100% for primary NSCLC tumors, with a median SUVmax of 8.74. While the SUVmax from [^68^Ga]Ga‐Trivehexin was lower than that of [^18^F]FDG, [^68^Ga]Ga‐Trivehexin showed a superior TLR of 8.82, enabling clearer tumor visualization. Overall, [^68^Ga]Ga‐Trivehexin and [^18^F]FDG had comparable detection efficacy for the diagnosis of NSCLC primary tumors.

Precise preoperative mediastinal lymph node staging is essential for the optimal surgical strategy selection and clinical outcome for resectable NSCLC.^[^ ^29^ ^]^ [^18^F]FDG PET/CT is considered more effective than CT alone for N‐staging in lung cancer.^[^ ^30^ ^]^ However, inflammatory lymph nodes can lead to false‐positive findings on [^18^F]FDG PET/CT.^[^ ^31^, ^32^ ^]^ A previous study revealed a sensitivity and specificity of 75% and 85%, respectively, for N‐staging from a per‐node station analysis; when factors including lymph node calcification and symmetrical distribution pattern were considered, the diagnostic specificity increased to 96%, but the sensitivity decreased to 66%.^[^ ^33^ ^]^ Our study demonstrated that [^68^Ga]Ga‐Trivehexin exhibited higher specificity (93.8% vs 62.5%) and accuracy (91.2% vs 64.2%) compared with [^18^F]FDG. There were 42 false‐positive and 7 false‐negative lymph nodes on [^18^F]FDG, whereas there were only 7 false‐positive and 5 false‐negative lymph nodes on [^68^Ga]Ga‐Trivehexin PET/CT. Most of the false‐positive lymph nodes in the mediastinum and bilateral hilar areas were caused by inflammation or infection and were symmetrically distributed on [^18^F]FDG PET/CT. The reason for false‐positive nodes on [^68^Ga]Ga‐Trivehexin may be because of macrophages engulfing necrotic debris of cancer cells that had not been completely degraded and entering the lymph node. It is important to note that both tracers failed to detect micrometastases in lymph nodes with a short diameter of less than 5 mm, which may be associated with the limited spatial resolution of PET. Overall, [^68^Ga]Ga‐Trivehexin shows great potential in detecting lymph node metastasis and may provide earlier surgical opportunities for patients with resectable NSCLC.

Approximately 11%–36% of patients with NSCLC show distant metastases at initial diagnosis, with common metastatic sites consisting of bone, lung, brain, adrenal gland, liver, and soft tissue.^[^ ^4^, ^34^ ^]^ Our study found no significant difference between [^68^Ga]Ga‐Trivehexin and [^18^F]FDG PET/CT in the evaluation of bone, pleural, and liver metastases. However, the TLR on [^68^Ga]Ga‐Trivehexin was higher than that of [^18^F]FDG in bone and liver metastases, resulting in a distinct contour of the lesion. Notably, we observed that the physiological uptake of [^68^Ga]Ga‐Trivehexin in the normal brain parenchyma was extremely low because there was no integrin αvβ6 expression in normal brain parenchyma, enabling easier detection of brain metastases compared with [^18^F]FDG. Berghoff and colleagues demonstrated that integrin αvβ6 is involved in the brain metastatic cascade in NSCLC.^[^ ^35^ ^]^ The authors performed immunostaining of integrin αvβ6 in 191 brain metastasis specimens from 191 lung cancer patients (172 NSCLC and 19 SCLC); the results revealed that 53.9% (103/191) samples showed integrin αvβ6 expression, which was more likely to be observed in NSCLC (57%) than in SCLC (26.3%). Moreover, the expression of integrin αvβ6 in the primary tumor was associated with its expression in brain metastasis (*p* = 0.034).^[^ ^35^ ^]^ These findings provide strong evidence for the detection of brain metastases using [^68^Ga]Ga‐Trivehexin PET in NSCLC. In the current study, because of the excellent diagnostic performance with [^68^Ga]Ga‐Trivehexin PET/CT, therapeutic regimens were changed in 14 patients. These findings demonstrate the potential advantages and prospects of [^68^Ga]Ga‐Trivehexin in the clinical staging and therapeutic management of NSCLC.

Integrin αvβ3, an important member of the integrin family, was the first integrin shown to be closely related to tumor angiogenesis and highly expressed in tumor neovascularization and cancer cells. Integrin αvβ3 has been extensively studied from the bench to clinical applications as an attractive biomarker for cancer imaging, as it contributes to every step of tumorigenesis and tumor progression.^[^ ^36^ ^]^ In the past two decades, several αvβ3‐targeted radiopharmaceuticals have been developed and translated into clinical studies for detecting malignant tumors, including NSCLC.^[^ ^37^ ^]^ Kang et al. demonstrated that the αvβ3‐targeted probe [^68^Ga]Ga‐NOTA‐E[PEG_4_‐c(RGDfk)]_2_ (^68^Ga‐Alfatide II) could differentiate between NSCLC (*n* = 21) and lung tuberculosis (*n* = 13).^[^ ^38^ ^]^ The diagnostic sensitivity and specificity of ^68^Ga‐Alfatide II were 85.7% and 84.6%, respectively, for detecting NSCLC primary tumors, with SUVmax of 3.83 for the primary tumor and 2.29 for tuberculosis (*p* = 0.0001). For lymph node metastasis, ^68^Ga‐Alfatide II exhibited superior detection specificity (100% vs 66.7%) and a slightly lower sensitivity (75% vs 87.5%) compared with [^18^F]FDG. For distant metastasis, ^68^Ga/^18^F‐Alfatide II showed advantages for detecting brain metastasis because of the extremely low background accumulation in normal brain parenchyma.^[^ ^38^, ^39^ ^]^ However, ^68^Ga‐Alfatide II failed to detect most bone and liver metastases.^[^ ^38^ ^]^ Similar to ^68^Ga‐Alfatide II, integrin αvβ6‐targeted [^68^Ga]Ga‐Trivehexin shows significant advantages in identifying lymph node and brain metastases in NSCLC. The difference between [^68^Ga]Ga‐Trivehexin and ^68^Ga‐Alfatide II is that [^68^Ga]Ga‐Trivehexin can also sensitively detect distant metastatic lesions involving the pleura, bone, liver, and soft tissues.

A previous study demonstrated that the integrin αvβ3‐specific radiotracer [^68^Ga]Ga‐DOTA‐RGD_2_ could non‐invasively quantify the expression level of αvβ3 between NSCLC (*n* = 21) and SCLC (*n* = 10), with a distinctly higher uptake value of [^68^Ga]Ga‐DOTA‐RGD_2_ in NSCLC primary tumors than in SCLC lesions (SUVmax, 3.83 vs 2.05; *p* < 0.0001). Moreover, histopathological evidence revealed positive αvβ3 expression in NSCLC but negligible expression in SCLC.^[^ ^40^ ^]^ In our cohort, four cases were confirmed as SCLC by biopsy, including four true‐positive patients on [^18^F]FDG but three true‐positive cases and one false‐negative case on [^68^Ga]Ga‐Trivehexin (Figure S3B, Supporting Information). Inter‐ and intra‐tumor heterogeneity was found in the lesions of SCLC on [^68^Ga]Ga‐Trivehexin with SUVmax of 3.12–8.57 and SUVmean of 1.87–4.17 in primary tumors. The median SUV from [^68^Ga]Ga‐Trivehexin in NSCLC primary tumors was higher compared with that in SCLC (SUVmax, 8.74 vs 4.98, *p* = 0.059; SUVmean, 5.09 vs 2.64, *p* = 0.024), suggesting low or no expression of integrin β6 in SCLC. In line with the fact that the expression of integrin β6 is mainly restricted to epithelial‐derived carcinomas and not neuroendocrine tumors, we presume that [^68^Ga]Ga‐Trivehexin is not a suitable radiopharmaceutical for imaging SCLC.

Studies have shown that integrin αvβ6 is an important and independent prognostic biomarker that is significantly associated with poor patient outcomes for NSCLC.^[^ ^16^ ^]^ Elayadi et al. reported that 54% of lung cancer samples had positive αvβ6 expression, and NSCLC showed more integrin β6 expression than SCLC, indicating the potential application of integrin β6 as a biomarker for NSCLC.^[^ ^16^ ^]^ Meijer et al. demonstrated that integrin αvβ6 expression was significantly higher in the tumor than in the surrounding normal lung tissues in 15 lung adenocarcinoma cases (*p* = 0.006), but there was no significant difference in 15 lung squamous cell carcinoma cases (*p* = 0.705).^[^ ^41^ ^]^ In the current study, all examined tumor tissues and metastatic lymph nodes showed integrin β6 expression with varying degrees, and a positive rate of 100%. Moreover, [^68^Ga]Ga‐Trivehexin uptake was positively associated with intense integrin β6 expression, higher histological grading, and advanced clinical staging in NSCLC. For high‐grade NSCLC, including poor‐differentiated adenocarcinoma, sarcomatoid carcinoma, adenosquamous carcinoma, and large cell neuroendocrine carcinoma, the positive percentage was higher than 50%; in contrast, the percentage was relatively low (only ≈5%–30%) in most squamous cell carcinomas and well‐differentiated adenocarcinoma. Previous studies have reported an increased intensity of integrin β6 expression in tumor cells adjacent to the tumor stroma in pancreatic ductal adenocarcinoma.^[^ ^42^ ^]^ We found that integrin β6 staining showed the following key characteristics in NSCLC: i) the tumor cells adjacent to the tumor stroma exhibited strong integrin β6 expression compared with central tumor cells far away from tumor stroma; ii) in a cancer nest, the tumor cells in the outer layer showed stronger integrin β6 expression than those in the inner layer; iii) the intensity of integrin β6 immunostaining was increased at the edge of invasive tumors; and iv) in some tumor samples, integrin β6 expression was also observed to a lesser extent in the reactive proliferative alveolar epithelium in the normal lung tissue surrounding the tumor. This distribution pattern of integrin β6 expression may be related to the tumor microenvironment, which promotes tumor occurrence, proliferation, invasion, and metastasis. Together, these data indicate that integrin αvβ6 may provide new significant opportunities for targeted imaging and radioligand therapy in NSCLC in the near future.

Integrin αvβ6 has multiple pro‐tumorigenic effects, with roles in several different signaling pathways and functions in accelerating tumor progression.^[^ ^43^ ^]^ For instance, integrin αvβ6 can induce the activation and release of TGFβ1 from the latent TGFβ complexes and subsequently activate TGFβ/SMAD signaling pathway to aid cancer metastasis by positively regulating the EMT process. Additionally, integrin αvβ6 activates MMPs activity and increases extracellular matrix degradation to promote EMT and the migration of post‐EMT cells on fibronectin in cancers.^[^ ^43^, ^44^ ^]^ Integrin αvβ6 promotes lung cancer cell proliferation, migration, and invasion by impairing the expression of MMP2 and MMP9 and induces tumor growth by inhibiting cancer cell apoptosis. Integrin αvβ6 was also shown to stimulate cancer metastasis, which was partly from the upregulation of interleukin‐8‐mediated signaling.^[^ ^45^ ^]^ In our study, we performed immunohistochemistry of multiple proteins (TGFβ1, Smad2, fibronectin, vimentin, MMP9, and Ki‐67) in tumor samples to explore the possible mechanism of integrin αvβ6 in NSCLC. The tumor uptake on [^68^Ga]Ga‐Trivehexin distinctly correlated with TGFβ1 and Ki‐67, except for integrin αvβ6. However, there was no relationship between tumor uptake on [^68^Ga]Ga‐Trivehexin and Smad2, fibronectin, vimentin, or MMP9. One possible reason for this result may be the limited sample size. Therefore, the role of integrin αvβ6 in the carcinogenesis and metastasis of NSCLC still needs further in‐depth research.

This study has several limitations. First, obtaining histopathologic validation for all distant metastatic lesions in advanced lung cancer was not feasible or ethical, so typical imaging findings and follow‐up data were used as the reference standard. Second, the limited number of distant metastases may have affected the representativeness of the diagnostic performance results between the two imaging agents. Third, the study only included solid tumors, excluding ground glass nodules, which may not reflect the full scope of the diagnostic performance of [^68^Ga]Ga‐Trivehexin in NSCLC. Further prospective studies with larger cohorts are warranted to confirm our research findings.

## 4 Conclusion

Integrin αvβ6 is a potential and promising biomarker for both diagnosis and therapy in malignancies with intensive integrin β6 expression. Our study demonstrated that the αvβ6‐targeted radiotracer [^68^Ga]Ga‐Trivehexin PET/CT exhibited significantly better detection performance for identifying mediastinal lymph nodes and brain metastases compared with [^18^F]FDG and comparable diagnostic performance for the detection of primary tumors and other distant metastases in NSCLC. Additionally, there was a significant positive correlation between tumor [^68^Ga]Ga‐Trivehexin uptake and integrin β6 expression. Therefore, [^68^Ga]Ga‐Trivehexin PET/CT is a noninvasive and promising imaging diagnostic tool that can provide accurate clinical staging and guide the selection of treatment strategies for patients with NSCLC.

## 5 Experimental Section

### Participants

This single‐center prospective study was approved by the Medical Ethics Committee of Zhongnan Hospital of Wuhan University and registered at ClinicalTrials.gov (NCT05835570). A total of 79 participants with suspected lung cancer or pathologically confirmed NSCLC at our hospital were enrolled between January 2023 and September 2024. All participants provided written informed consent. The inclusion criteria were as follows: i) clinically suspected lung cancer or confirmed NSCLC; ii) completion of both [^18^F]FDG and [^68^Ga]Ga‐Trivehexin PET/CT examinations within 1 week; and iii) no prior antitumor treatments (e.g., chemotherapy and radiotherapy) before PET/CT imaging. The exclusion criteria were as follows: i) severe liver and kidney dysfunction; ii) lack of pathological confirmation; iii) presence of second primary malignant tumors; iv) pulmonary ground glass nodule; and v) lung lesions confirmed not to be NSCLC.

### Preparation of [^68^Ga]Ga‐Trivehexin

[^68^Ga]Ga‐Trivehexin was prepared for research purposes using the iQS‐TS automated synthesis module (Isotopen Technologien München AG) following a previously validated protocol.^[^ ^46^ ^]^ Briefly, ^68^GaCl_3_ was eluted from the ^68^Ge/^68^Ga generator using 4 mL of 0.05 M hydrochloric acid into a reaction vessel containing a mixture of precursor (30 µg) and sodium acetate buffer (1 mL). After heating at 95 °C for 10 min, a pre‐activated Sep‐Pak C18 cartridge (Waters Corporation) was used for purification of the mixture, and a 0.22 µm syringe filter (Merck KGaA) was used for sterilization. [^68^Ga]Ga‐Trivehexin was obtained as a colorless and transparent solution with a radiochemical yield of 71.3% ± 1.7% and radiochemical purity exceeding 99% as demonstrated by high‐performance liquid chromatography (Waters Corporation).

### Imaging Acquisition

All [^18^F]FDG and [^68^Ga]Ga‐Trivehexin PET/CT scans were completed within 1 week for each participant. Standard [^18^F]FDG PET/CT was first performed. The participants were required to fast for at least 6 h, and blood glucose levels were required to be less than 11.0 mmol L^−1^ prior to the scan. PET/CT scans (Biograph mCT; Siemens Healthineers) were performed ≈60 min after an i.v. injection of the [^18^F]FDG tracer, with an injection dose of 3.70–5.55 MBq kg^−1^. Unenhanced CT (tube voltage, 120 keV; tube current, 30–100 mA, auto mA mode; pitch, 0.8; slice thickness, 3.00 mm; rotation speed, 0.8 s) was performed for attenuation correction and anatomical localization, with the scan range from the top of the skull to the upper thigh. PET acquisition was subsequently performed in 3D mode with 1.5 min per bed position. Six to eight beds were acquired depending on the participant's height. PET data were reconstructed using the ordered subset expectation maximization method and transferred to the MEMRS‐NM Workstation (MedEx Technology Limited Corporation, Beijing, China) for imaging analysis.

For [^68^Ga]Ga‐Trivehexin PET/CT, no special preparation was required. Approximately 45–60 min after an i.v. administration of [^68^Ga]Ga‐Trivehexin with a dose of 1.85–2.22 MBq kg^−1^, PET/CT scan was performed as described above, except the PET acquisition time was 2.5 min per bed. No adverse events or abnormal vital signs were observed from the beginning of the injection of [^68^Ga]Ga‐Trivehexin until 3 h after completing the examination.

### Image Interpretation

All PET/CT images were independently reviewed by two board‐certified nuclear medicine physicians (C.L. and Y.T.) who were blinded to the pathological information. On visual analysis, any focal tracer accumulation higher than the uptake of mediastinal blood pool was considered a positive lesion; physiological uptake and benign lesions were excluded. For semi‐quantitative analysis, the SUVmax was obtained by manually drawing the volume of interest around the lesion. The SUVmean, metabolic tumor volume (MTV), and [^68^Ga]Ga‐Trivehexin‐derived ITV of the lesions were measured using 40% of SUVmax as the threshold. TLG was calculated as MTV × SUVmean, and [^68^Ga]Ga‐Trivehexin‐derived TLI was calculated as ITV × SUVmean, respectively. TBR and TLR were determined as the SUV of the lesion divided by the SUV of the mediastinal blood pool and normal hepatic parenchyma, respectively. TBR_b_ was calculated to evaluate brain metastases, taking normal brain parenchyma as background. Any diagnostic discrepancies between the two reviewers were resolved by discussion. The International Association for the Study of Lung Cancer lymph node classification criteria (station 2L/R, 3, 4L/R, 5, 6, 7, 8, 9L/R, 10–12L/R) were used to assess lymph node metastasis.^[^ ^47^ ^]^

### Reference Standards

All primary tumors and lymph node metastases were determined by surgical pathology or fine needle aspiration biopsy. For distant metastatic lesions without pathological findings, typical CT/MRI imaging features or follow‐up results for more than 3 months were used as reference criteria. For instance, i) osteolytic, osteogenic, or mixed bone destruction with or without soft tissue mass is considered the typical imaging manifestation of bone metastasis;^[^ ^48^ ^]^ ii) brain metastases are typically solid or ring‐enhancing nodules with peripheral cerebral edema on MRI;^[^ ^49^ ^]^ and iii) ring enhancement in early contrast‐enhanced CT and delayed central region enhancement in the equilibrium phase are typical features of liver metastases.^[^ ^50^ ^]^

### Histological Analysis

H&E and immunohistochemistry staining were performed on available excisional biopsy or needle biopsy samples. Formalin‐fixed and paraffin‐embedded blocks were cut into 4‐µm sections for H&E and immunohistochemistry staining. Immunohistochemistry was performed on three different automated immunohistochemical instruments following our established protocols. We used integrin β6 expression to indicate integrin αvβ6 expression because the integrin β6 subunit only dimerizes with the ubiquitous αv subunit. The antibodies selected for immunohistochemistry staining included anti‐integrin β6 (1:200 dilution, rabbit monoclonal, #95153; CST), anti‐TGFβ1 (1:200 dilution, rabbit monoclonal, ab215715; abcam), anti‐Smad2 (1:100 dilution, rabbit monoclonal, ab188334; abcam), anti‐fibronectin (1:250 dilution, rabbit monoclonal, ab32419; abcam), anti‐MMP9 (1:500 dilution, rabbit monoclonal, ab194316; abcam), anti‐vimentin (ready‐to‐use, mouse monoclonal, UMAB159; Zhongshanjinqiao), anti‐Ki‐67 (ready‐to‐use, mouse monoclonal, MIB1; Zhongshanjinqiao), anti‐CD34 (ready‐to‐use, mouse monoclonal, QBEnd10; Dako Omnis), anti‐CD4 (ready‐to‐use, mouse monoclonal, 4B12; Dako Omnis), and anti‐CD8 (ready‐to‐use, mouse monoclonal, C8/144B; Dako Omnis). All the slides were counterstained with 3,3′‐diaminobenzidine (DAB). Vimentin and Ki‐67 staining were performed on a Leica BOND‐MAX autostainer; staining for the other markers was performed on a DAKO AutostainerLink 48. Nine samples were double‐stained for integrin β6 (staining cancer cells) and CD34 (highlighting vascular endothelial cells within the tumor stroma) on a BenchMark ULTRA PLUS autostainer (Roche, AZ, USA). For the double staining, counterstaining was performed using DAB for CD34 (brown staining) and new fuchsin for integrin β6 (rose‐red coloration). The senior pathologist (Q.C.) observed all the slides under a light microscope (Olympus BX‐53, Tokyo, Japan).

The samples were scored semi‐quantitatively by assessing staining intensities and the proportions of positive cancer cells. For integrin β6 single staining, the intensity was scored as follows: 0, negative; 1, weak (+, yellow); 2, medium (++, brown); and 3, strong (+++, dark brown). For the double staining, integrin β6 staining showing rose‐red was scored as follows: 0, negative; 1, weak (+); 2, medium (++); and 3, strong (+++). The proportion of positively stained cells was scored as follows: 0 (negative), 1 (1%–25%), 2 (25%–50%), 3 (50%–75%), and 4 (75%–100%). The integrated score (IS) was calculated as the intensity score multiplied by the proportion scores, with a range from 0–12. The final immunostaining scores were stratified as follows: 0 (IS = 0), 1 (IS = 1–4), 2 (IS = 5–8), and 3 (IS = 9–12).

### Statistical Analyses

Statistical analyses were performed using SPSS software (version 26.0, IBM) and GraphPad Prism 9.5. Categorical data are presented as frequency (percentages). Continuous variables are expressed as mean ± standard deviation if data follow a normal distribution; otherwise, median (interquartile range [IQR]) is presented. The Wilcoxon signed‐rank test or Mann‐Whitney *U* test was used to compare two continuous variables with non‐normal distributions; otherwise, the independent sample *t‐*test was used. Diagnostic sensitivity, specificity, positive predictive value, negative predictive value, and accuracy were calculated. McNemar's test was used to compare the diagnostic performance between the two imaging agents. ROC analysis was used to evaluate the diagnostic performance of each parameter for detecting metastatic lymph nodes. The AUC values was compared using MedCalc software (version 20.2). Spearman correlation coefficient was used to assess the association of tumor tracer uptake with immunohistochemistry, histological grading, and clinical staging. A *p*‐value < 0.05 indicated statistical significance.

### Ethics statement

This single‐center prospective study was approved by the Medical Ethics Committee of Zhongnan Hospital of Wuhan University and registered at ClinicalTrials.gov (NCT05835570).

## Conflict of Interest

The authors declare no potential conflicts of interest.

## Author Contributions

Y.H. and J.H. contributed to the design of the clinical trial. H.W. and L.L. contributed to participant enrollment, clinical data collection, and statistical analysis. C.L. and Y.T. contributed to reviewing PET/CT images and the interpretation of results. Z.X. and Y.H. contributed to the preparation and quality control of [^68^Ga]Ga‐Trivehexin. J.Z. was responsible for PET/CT scan and post‐processing of the images. Q.C. performed immunohistochemical staining, scoring, photography, and analyses. H.W. and C.L. drafted the initial version of the manuscript. Y.H., H.W., C.L., L.L., Q.C., and J.H. were responsible for the revision of the manuscript. All authors read and approved the final manuscript. H.W., C.L., and L.L. contributed equally to this work.

## Supporting information



Supporting Information

## Data Availability

The data that support the findings of this study are available from the corresponding author upon reasonable request.
