# Supplementary material for: Integrin αvβ6‐Targeted PET/CT Imaging of Non‐Small Cell Lung Cancer with [68Ga]Ga‐Trivehexin: Improved Preoperative Lymph Node Staging and Association with Immunohistochemistry
Source: Adv Sci (Weinh). 2025 Sep 3;12(44):e08225. doi: 10.1002/advs.202508225 (PMC12667533; doi:10.1002/advs.202508225)
Supplement: Supplementary file 1 — Supporting Information [file ADVS-12-e08225-s001.docx]

**Supporting Information**

**Title:** Integrin αvβ6-targeted PET/CT Imaging of Non-Small Cell Lung Cancer with [^68^Ga]Ga-Trivehexin: Improved Preoperative Lymph Node Staging and Association with Immunohistochemistry

**Authors:** Huiqin Wu^1#^, Chongjiao Li^1#^, Ling Li^1#^, Yueli Tian^1^, Zhiwei Xiao^1^, Yiwei Huang^1^, Juan Zhong^1^, Jianying Huang^3^*, Qiongrong Chen^2^*, Yong He^1^*

^#^ Huiqin Wu, Chongjiao Li, and Ling Li contributed equally to this work.

**Affiliations:**

^1^ Department of Nuclear Medicine, Zhongnan Hospital of Wuhan University, Wuhan, China; ^2^ Department of Pathology, Zhongnan Hospital of Wuhan University, Wuhan, China; ^3^ Clinical Trial Center, Zhongnan Hospital of Wuhan University, Wuhan, China

***Correspondence to:**

Prof. Yong He, Email: [heyong@whu.edu.cn](mailto:heyong@znhospital.cn);

Prof. Qiongrong Chen, Email: [qiongrongchen@whu.edu.cn](mailto:qiongrongchen@whu.edu.cn);

Prof. Jianying Huang, Email: huangjianying@znhospital.cn

**Address:** Department of Nuclear Medicine, Zhongnan Hospital of Wuhan University, No. 169 East Lake Road, Wuchang District, Wuhan, Hubei Province 430071, China.

| **Supplementary Table S1 Participants characteristics** | |
| --- | --- |
| **Characteristics** | **Value (%)** |
| **No. of participants** | 58 |
| **Age (years, range)** | 45–90 |
| Mean ± SD | 62.6 ± 8.4 |
| **Sex** |  |
| Men | 45 (77.6) |
| Women | 13 (22.4) |
| **Smoking history** |  |
| Yes | 27 (46.6) |
| No | 31 (53.4) |
| **Primary tumor size (mm, range)** | 11.0–110.0 |
| Median (IQR) | 36.5 (25.0, 50.3) |
| **Location of primary tumor** |  |
| Left upper lobe | 17 (29.3) |
| Left lower lobe | 12 (20.7) |
| Right upper lobe | 11 (19.0) |
| Right middle lobe | 4 (6.9) |
| Right lower lobe | 14 (24.1) |
| **Pathological type** |  |
| Adenocarcinoma | 30 (51.7) |
| Squamous cell carcinoma | 19 (32.8) |
| Large cell neuroendocrine carcinoma | 4 (6.9) |
| Pulmonary mucinous adenocarcinoma | 2 (3.5) |
| Adenosquamous carcinoma | 1 (1.7) |
| Pulmonary sarcomatoid carcinoma | 1 (1.7) |
| Undefined type | 1 (1.7) |
| **TNM stage** |  |
| Ⅰ | 15 (25.9) |
| Ⅱ | 10 (17.2) |
| Ⅲ | 14 (24.1) |
| Ⅳ | 19 (32.8) |
| Notes. Unless otherwise indicated, values are number and percentage.  IQR, interquartile range; TNM, tumor-node-metastasis (the 8^th^ edition of the American Joint Committee on Cancer (AJCC) staging system). | |

| **Supplementary Table S2 Comparison of tracer uptake in primary tumor on [68Ga]Ga-Trivehexin and [18F]FDG PET/CT in non-small cell lung cancer** | | | | | | | | | | | | |
| --- | --- | --- | --- | --- | --- | --- | --- | --- | --- | --- | --- | --- |
| **Group** | **[68Ga]Ga-Trivehexin PET/CT** | | | | | **[18F]FDG PET/CT** | | | | | | |
|  | **SUVmax** | **TBR** | **TLR** | **ITV** | **TLI** | **SUVmax** | **TBR** | | **TLR** | | **MTV** | **TLG** |
| **All (***n* = 58) | 8.74 (5.78, 12.68) | 8.07 (5.36, 10.38) | 8.82 (6.10, 11.44) | 14.07 (5.64, 32.20) | 67.38 (19.79, 176.31) | 15.12 (11.00, 18.86) | 7.91 (4.85, 11.19) | | 5.98 (4.85, 8.57) | | 10.33 (3.87, 30.69) | 99.11 (20.00, 308.58) |
| **Age** | | | | | | | |  | |  |  |  |
| ≤ 60 (*n* = 27) | 8.27 (5.55, 13.33) | 7.92 (5.58, 11.14) | 8.23 (5.60, 11.41) | 11.51 (4.38, 35.07) | 65.44 (14.70, 183.94) | 16.99 (12.25, 18.73) | 9.41 (5.48, 12.53) | | 6.73 (4.50, 9.44) | | 9.12 (2.62, 37.09) | 110.35 (18.51, 310.68) |
| > 60 (*n* = 31) | 8.91 (6.27, 12.50) | 8.22 (5.18, 9.98) | 9.06 (7.23, 11.92) | 14.63 (6.77, 29.93) | 69.32 (34.41, 173.77) | 13.12 (8.19, 19.28) | 6.43 (4.12, 9.82) | | 5.04 (3.18, 7.78) | | 11.68 (3.98, 29.86) | 88.74 (32.01, 300.44) |
| *p* value | 0.895 | 0.773 | 0.714 | 0.703 | 0.761 | 0.215 | **0.049** | | 0.054 | | 0.821 | 0.809 |
| **Sex** | | | | | | | | | | | | |
| Women (*n* = 13) | 10.29 (6.06, 13.24) | 8.22 (4.81, 11.84) | 11.41 (6.90, 14.67) | 7.66 (5.13, 14.65) | 48.74 (16.81, 79.88) | 12.58 (5.64, 17.65) | 4.94 (2.92, 8.93) | | 4.18 (2.42, 7.13) | | 6.90 (2.49, 13.07) | 50.95 (17.42, 116.84) |
| Men (*n* = 45) | 8.57 (5.77, 12.34) | 7.92 (5.38, 10.40) | 8.80 (5.81, 10.79) | 16.65 (5.71, 37.52） | 83.78 (22.97, 193.17) | 16.23 (11.50, 19.46) | 8.49 (5.38, 11.36) | | 6.05 (4.32, 8.80) | | 11.81 (4.01, 35.22) | 112.31 (26.36, 356.93) |
| *p* value | 0.450 | 0.948 | 0.236 | 0.103 | 0.259 | 0.138 | **0.043** | | 0.064 | | 0.222 | 0.119 |
| **Smoking history** | | | | | | | | | | | | |
| Yes (*n* = 27) | 8.34 (5.55, 10.98) | 7.34 (5.18, 10.24) | 8.32 (5.60, 9.94) | 18.68 (7.66, 75.54) | 119.25 (25.86, 332.91) | 16.23 (11.36, 18.73) | 9.42 (5.23, 12.38) | | 7.08 (4.39, 8.87) | | 12.77 (4.68, 46.81) | 118.73 (32.01, 422.90) |
| No (*n* = 31) | 8.91 (6.27, 13.09) | 8.74 (5.42, 11.14) | 9.76 (6.97, 11.92) | 9.06 (4.74, 25.18) | 53.66 (17.81, 147.22) | 14.81 (8.19, 20.15) | 7.00 (4.13, 9.96) | | 5.66 (3.40, 7.78) | | 8.43 (2.62, 19.47) | 60.26 (16.33, 197.74) |
| *p* value | 0.450 | 0.370 | 0.204 | 0.084 | 0.239 | 0.809 | 0.221 | | 0.227 | | 0.173 | 0.204 |
| **Tumor size** | | | | | | | | | | | | |
| ≤ 3cm (*n* = 22) | 5.67 (4.17, 8.55) | 5.30 (4.14, 7.54) | 6.47 (4.41, 9.19) | 4.84 (3.25, 8.49) | 16.26 (8.26, 39.56) | 11.97 (7.12, 16.42) | 5.61 (3.58, 8.59) | | 4.37 (2.87, 6.16) | | 2.60 (1.63, 4.84) | 16.77 (8.01, 43.74) |
| > 3cm (*n* = 36) | 10.82 (7.76. 13.37) | 9.45 (6.58, 11.12) | 9.79 (8.09, 12.25) | 24.37 (11.80, 50.28) | 132.96 (66.41, 296.37) | 17.57 (12.31, 21.56) | 9.54 (5.85, 12.49) | | 7.13 (4.89, 9.46) | | 18.84 (9.18, 46.60) | 221.73 (97.47, 495.06) |
| *p* value | **< 0.001** | **< 0.001** | **0.001** | **< 0.001** | **< 0.001** | **0.003** | **0.002** | | **0.002** | | **< 0.001** | **< 0.001** |
| **TNM stage** | | | | | | | | | | | | |
| Ⅰ–Ⅱ (*n* = 26) | 7.56 (4.95, 9.88) | 6.20 (5.12, 9.60) | 8.01 (5.39, 11.28) | 6.62 (4.01, 16.94) | 35.89 (10.91, 73.01) | 12.29 (6.26, 17.11) | 5.90 (2.94, 8.71) | | 4.75 (2.62, 6.67) | | 4.74 (1.96, 12.67) | 26.47 (10.48, 107.56) |
| Ⅲ–Ⅳ (*n* = 32) | 10.54 (7.56, 13.02) | 9.16 (6.03, 10.43) | 9.14 (7.27, 12.25) | 22.08 (9.53, 49.90) | 130.95 (53.58, 274.30) | 17.57 (12.52, 20.45) | 9.74 (5.78, 12.49) | | 7.65 (4.74, 9.46) | | 18.74 (8.20, 49.14) | 204.27 (89.40, 609.47) |
| *p* value | **0.019** | 0.065 | 0.133 | **0.001** | **< 0.001** | **0.002** | **0.002** | | **0.003** | | **0.001** | **< 0.001** |
| **Pathology type** | | | | | | | | | | | | |
| ADC (*n* = 29) | 8.27 (5.12, 11.95) | 7.16 (5.29, 9.82) | 8.98 (5.81, 11.73) | 8.75 (3.87, 18.38) | 48.74 (14.52, 87.11) | 12.58 (7.15, 18.61) | 6.43 (3.85, 9.89) | | 5.52 (3.16, 7.86) | | 6.90 (2.34, 18.84) | 52.46 (15.68, 160.53) |
| Non-ADC (*n* = 29) | 9.28 (6.46, 12.76) | 8.54 (5.38, 10.84) | 8.80 (6.61, 11.00) | 18.71 (8.48, 45.20) | 127.57 (30.14, 243.36) | 16.47 (12.60, 19.27) | 9.35 (5.90, 11.36) | | 6.65 (4.75, 9.10) | | 15.56 (6.07, 35.22) | 132.56 (40.17, 356.93) |
| *p* value | 0.304 | 0.231 | 0.779 | **0.014** | **0.017** | 0.062 | **0.025** | | **0.023** | | **0.048** | **0.022** |
| Note. SUV, standardized uptake value; TBR, tumor-to-background (mediastinal blood pool) ratio; TLR, tumor-to-liver parenchyma ratio; TNM, tumor-node-metastasis; ADC, adenocarcinoma; ITV, integrin β6 expression tumor volume；TLI, total lesion integrin β6 expression; MTV, metabolic tumor volume；TLG, total lesion glycolysis.  **p* < 0.05 represents statistical difference. | | | | | | | | | | | | |

| **Supplementary Table S3 Comparison of diagnostic performance of [^68^Ga]Ga-Trivehexin and [^18^F]FDG PET/CT for the detection of lymph node (LN) metastasis in non-small cell lung cancer (lesion-based analysis)** | | | | | | | | | | | | | | | | | | |
| --- | --- | --- | --- | --- | --- | --- | --- | --- | --- | --- | --- | --- | --- | --- | --- | --- | --- | --- |
| **Location of LN** | **[^68^Ga]Ga-Trivehexin PET/CT** | | | | | | | | | **[^18^F]FDG PET/CT** | | | | | | | | |
|  | **TP** | **F**  **P** | **FN** | **T**  **N** | **Sensitivity (%)** | **Specificity**  **(%)** | **PPV**  **(%)** | **NPV**  **(%)** | **Accuracy**  **(%)** | **TP** | **FP** | **FN** | **T**  **N** | **Sensitivity (%)** | **Specificity**  **(%)** | **PPV**  **(%)** | **NPV**  **(%)** | **Accuracy**  **(%)** |
| **Station (2L)** | 2 | 0 | 0 | 1 | 100 (2/2) | 100 (1/1) | 100 (2/2) | 100 (1/1) | 100 (3/3) | 2 | 0 | 0 | 1 | 100 (2/2) | 100 (1/1) | 100 (2/2) | 100 (1/1) | 100 (3/3) |
| **Station (2R)** | 1 | 0 | 0 | 17 | 100 (1/1) | 100 (17/17) | 100 (1/1) | 100 (17/17) | 100 (18/18) | 0 | 2 | 0 | 16 | NA | 88.9 (16/18) | NA | 100 (16/16) | 88.9 (16/18) |
| **Station (3)** | 0 | 0 | 0 | 4 | NA | 100 (4/4) | NA | 100 (4/4) | 100 (4/4) | 0 | 0 | 0 | 4 | NA | 100 (4/4) | NA | 100 (4/4) | 100 (4/4) |
| **Station (4L)** | 0 | 0 | 0 | 3 | NA | 100 (3/3) | NA | 100 (3/3) | 100 (3/3) | 0 | 2 | 0 | 1 | NA | 33.3 (1/3) | NA | 100 (1/1) | 33.3 (1/3) |
| **Station (4R)** | 5 | 3 | 0 | 46 | 100 (5/5) | 93.9 (46/49) | 62.5 (5/8) | 100 (46/46) | 94.4 (51/54) | 6 | 11 | 0 | 37 | 100 (6/6) | 77.1 (37/48) | 35.3 (6/17) | 100 (37/37) | 79.6 (43/54) |
| **Station (5)** | 1 | 1 | 7 | 16 | 12.5 (1/8) | 94.1 (16/17) | 50.0 (1/2) | 69.6 (16/23) | 68.0 (17/25) | 1 | 4 | 7 | 13 | 12.5 (1/8) | 76.5 (13/17) | 20.0 (1/5) | 65.0 (13/20) | 56.0 (14/25) |
| **Station (6)** | 4 | 0 | 1 | 5 | 80.0 (4/5) | 100 (5/5) | 100 (4/4) | 83.3 (5/6) | 90.0 (9/10) | 3 | 2 | 2 | 3 | 60.0 (3/5) | 60.0 (3/5) | 60.0 (3/5) | 60.0 (3/5) | 60.0 (6/10) |
| **Station (7)** | 3 | 2 | 1 | 67 | 75.0 (3/4) | 97.1 (67/69) | 60.0 (3/5) | 98.5 (67/68) | 95.9 (70/73) | 3 | 16 | 1 | 53 | 75.0 (3/4) | 76.8 (53/69) | 15.8 (3/19) | 98.2 (53/54) | 76.7 (56/73) |
| **Station (8)** | 0 | 0 | 0 | 1 | NA | 100 (1/1) | NA | 100 (1/1) | 100 (1/1) | 0 | 0 | 0 | 1 | NA | 100 (1/1) | NA | 100 (1/1) | 100 (1/1) |
| **Station (9L)** | 0 | 0 | 0 | 17 | NA | 100 (17/17) | NA | 100 (17/17) | 100 (17/17) | 0 | 0 | 0 | 17 | NA | 100 (17/17) | NA | 100 (17/17) | 100 (17/17) |
| **Station (9R)** | 0 | 0 | 0 | 6 | NA | 100 (6/6) | NA | 100 (6/6) | 100 (6/6) | 0 | 1 | 0 | 5 | NA | 83.3 (5/6) | NA | 100 (5/5) | 83.3 (5/6) |
| **Station (10L)** | 4 | 2 | 1 | 37 | 80.0 (4/5) | 94.9 (37/39) | 66.7 (4/6) | 97.4 (37/38) | 93.2 (41/44) | 4 | 10 | 3 | 27 | 57.1 (4/7) | 73.0 (27/37) | 28.6 (4/14) | 90.0 (27/30) | 70.5 (31/44) |
| **Station (10R)** | 1 | 0 | 2 | 16 | 33.3 (1/3) | 100 (16/16) | 100 (1/1) | 88.9 (16/18) | 89.5 (17/19) | 1 | 9 | 0 | 9 | 100 (1/1) | 50.0 (9/18) | 10.0 (1/10) | 100 (9/9) | 52.6 (10/19) |
| **Station (11–12L)** | 2 | 1 | 1 | 45 | 66.7 (2/3) | 97.8 (45/46) | 66.7 (2/3) | 97.8 (45/46) | 95.9 (47/49) | 1 | 2 | 2 | 44 | 33.3 (1/3) | 95.7 (44/46) | 33.3 (1/3) | 95.7 (44/46) | 91.8 (45/49) |
| **Station (11–12R)** | 1 | 0 | 2 | 24 | 33.3 (1/3) | 100 (24/24) | 100 (1/1) | 92.3 (24/26) | 92.6 (25/27) | 1 | 2 | 2 | 22 | 33.3 (1/3) | 91.7 (22/24) | 33.3 (1/3) | 91.7 (22/24) | 85.2 (23/27) |
| **Total** | 24 | 9 | 15 | 305 | 61.5 (24/39) | 97.1 (305/314) | 72.7 (24/33) | 95.3 (305/320) | 93.2 (329/353) | 22 | 61 | 17 | 253 | 56.4 (22/39) | 80.6 (253/314) | 26.5 (22/83) | 93.7 (253/270) | 77.9 (275/353) |
| Notes. TP, true-positive; FP, false-positive; FN, false-negative; TN, true-negative; PPV, positive predictive value; NPV, negative predictive value; NA, not applicable. | | | | | | | | | | | | | | | | | | |

| **Supplemental Table S4 Comparison of tracer uptake between non-metastatic and metastatic lymph nodes (LN) in participants with non-small cell lung cancer (lesion-based analysis)** | | | | | | |
| --- | --- | --- | --- | --- | --- | --- |
| **Parameters** | **[68Ga]Ga-Trivehexin** | | ***p* value** | **[18F]FDG** | | ***p* value** |
|  | **Non-metastatic LN** | **Metastatic LN** |  | **Non-metastatic LN** | **Metastatic LN** |  |
| **Station-based analysis** | | | | | | |
| SUVmax | 1.10 (0.99, 1.28) | 8.52 (4.71, 9.84) | **< 0.001** | 2.95 (2.09, 3.97) | 9.09 (7.09, 14.64) | **< 0.001** |
| TBR | 1.08 (0.99, 1.19) | 7.17 (4.41, 10.26) | **< 0.001** | 1.37 (1.13, 2.18) | 5.39 (3.28, 6.70) | **< 0.001** |
| TLR | 1.22 (0.94, 1.42) | 7.69 (4.74, 10.58) | **< 0.001** | 1.14 (0.92, 1.75) | 4.15 (2.62, 5.13) | **< 0.001** |
| **Lesion-based analysis** | | | | | | |
| SUVmax | 1.11 (1.00, 1.25) | 6.12 (2.78, 9.67) | **< 0.001** | 2.60 (1.97, 3.56) | 8.17 (5.81, 13.79) | **< 0.001** |
| TBR | 1.04 (0.96, 1.12) | 5.55 (2.41, 9.23) | **< 0.001** | 1.25 (1.07, 1.75) | 4.69 (2.25, 6.62) | **< 0.001** |
| TLR | 1.23 (0.96, 1.45) | 6.07 (2.62, 9.62) | **< 0.001** | 1.02 (0.86, 1.50) | 3.69 (2.00, 5.08) | **< 0.001** |
| Note. TBR, tumor-to-background (mediastinal blood pool) ratio; TLR, tumor-to-liver parenchyma ratio.  **p* < 0.05 represents statistical difference. | | | | | | |

| **Supplementary Table S5 ROC analysis of [68Ga]Ga-Trivehexin and [18F]FDG PET/CT for detecting lymph node metastasis in non-small cell lung cancer** | | | | | | | | | | | | | | | | | | | |
| --- | --- | --- | --- | --- | --- | --- | --- | --- | --- | --- | --- | --- | --- | --- | --- | --- | --- | --- | --- |
| **Parameters** | **AUC** | **95%CI** | | | ***p* value** | | **Cutoff value** | **Youden’s index** | | | **Sensitivity (%)** | **Specificity**  **(%)** | | **PPV (%)** | | | **NPV (%)** | | **Accuracy (%)** |
| **Station-based analysis** | | | | | | | | | | | | | | | | | | | |
| **[^68^Ga]Ga-Trivehexin** | | | | | | | | | | | | | | | | | | | |
| SUVmax | 0.980 | | 0.954-1.000 | **< 0.001** | | 2.350 | | | 0.923 | 95.5 (21/22) | | | 96.8 (92/95) | | 87.5 (21/24) | 98.9 (92/93) | | 96.6 (113/117) | |
| TBR | 0.961 | | 0.908–1.000 | **< 0.001** | | 2.141 | | | 0.923 | 95.5 (21/22) | | | 96.8 (92/95) | | 87.5 (21/24) | 98.9 (92/93) | | 96.6 (113/117) | |
| TLR | 0.973 | | 0.936–1.000 | **< 0.001** | | 2.380 | | | 0.913 | 95.5 (21/22) | | | 95.8 (91/95) | | 84.0 (21/25) | 98.9 (91/92) | | 95.7 (112/117) | |
| **[^18^F]FDG** | | | | | | | | | | | | | | | | | | | |
| SUVmax | 0.928 | | 0.860-0.996 | **< 0.001** | | 6.295 | | | 0.790 | 86.4 (19/22) | | | 92.6 (88/95) | | 73.1 (19/26) | 96.7 (88/91) | | 91.5 (107/117) | |
| TBR | 0.902 | | 0.827-0.977 | **< 0.001** | | 2.465 | | | 0.720 | 90.9 (20/22) | | | 81.1 (77/95) | | 52.6 (20/38) | 97.5 (77/79) | | 82.9 (97/117) | |
| TLR | 0.910 | | 0.839-0.982 | **< 0.001** | | 2.163 | | | 0.762 | 90.9 (20/22) | | | 90.0 (81/90) | | 58.8 (20/34) | 97.6 (81/83) | | 86.3 (101/117) | |
| **Lesion-based analysis** | | | | | | | | | | | | | | | | | | | |
| **[^68^Ga]Ga-Trivehexin** | | | | | | | | | | | | | | | | | | | |
| SUVmax | 0.963 | | 0.925–1.000 | **< 0.001** | | 1.750 | | | 0.862 | 88.5 (23/26) | | | 97.7 (211/216) | | 82.1 (23/28) | 98.6 (211/214) | | 96.7 (234/242) | |
| TBR | 0.938 | | 0.873–1.000 | **< 0.001** | | 2.102 | | | 0.827 | 84.6 (22/26) | | | 98.2 (212/216) | | 84.6 (22/26) | 98.2 (212/216) | | 96.7 (234/242) | |
| TLR | 0.951 | | 0.904–0.996 | **< 0.001** | | 2.008 | | | 0.834 | 84.6 (23/26) | | | 94.9 (205/216) | | 95.8 (23/34) | 98.6 (205/208) | | 94.2 (228/242) | |
| **[^18^F]FDG** | | | | | | | | | | | | | | | | | | | |
| SUVmax | 0.879 | | 0.785–0.973 | **< 0.001** | | 6.213 | | | 0.723 | 76.9 (20/26) | | | 95.4 (206/216) | | 66.7 (20/30) | 95.4 (206/212) | | 93.4 (226/242) | |
| TBR | 0.834 | | 0.715–0.952 | **< 0.001** | | 2.465 | | | 0.702 | 80.8 (21/26) | | | 89.4 (193/216) | | 87.5 (21/44) | 97.5 (193/198) | | 88.4 (214/242) | |
| TLR | 0.847 | | 0.735–0.960 | **< 0.001** | | 2.191 | | | 0.6715 | 80.8 (21/26) | | | 90.7 (196/216) | | 87.5 (21/41) | 97.5 (196/201) | | 89.7 (217/242) | |
| Note. ROC, receiver operating characteristic; AUC, area under the curve; CI, confidence interval; PPV, positive predictive value; NPV, negative predictive value; TBR, tumor-to-background (mediastinal blood pool) ratio; TLR, tumor-to-liver parenchyma ratio.  **p* < 0.05 represents statistical significance. | | | | | | | | | | | | | | | | | | | |

| **Supplementary Table S6 Comparison of diagnostic performance between [68Ga]Ga-Trivehexin and [18F]FDG PET/CT for the detection of distant metastasis in non-small cell lung cancer** | | | | | | | | | | |
| --- | --- | --- | --- | --- | --- | --- | --- | --- | --- | --- |
| **Location of lesions** | **TP** | **FP** | **FN** | **TN** | **Sensitivity (%)** | **Specificity (%)** | **PPV**  **(%)** | **NPV**  **(%)** | **Accuracy**  **(%)** |  |
| **Pleural metastasis** |  |  |  |  |  |  |  |  |  |  |
| [68Ga]Ga-Trivehexin | 45 | 0 | 4 | 0 | 91.8 (45/49) | NA | 100 (45/45) | NA | 91.8 (45/49) |  |
| [18F]FDG | 45 | 0 | 4 | 0 | 91.8 (45/49) | NA | 100 (45/45) | NA | 91.8 (45/49) |  |
| *p* value |  |  |  |  | NA | NA | NA | NA | NA |  |
| **Bone metastasis** |  |  |  |  |  |  |  |  |  |  |
| [68Ga]Ga-Trivehexin | 41 | 0 | 4 | 0 | 91.1 (41/45) | NA | 100 (41/41) | NA | 91.1 (41/45) |  |
| [18F]FDG | 45 | 0 | 0 | 0 | 100 (45/45) | NA | 100 (45/45) | NA | 100 (45/45) |  |
| *p* value |  |  |  |  | NA | NA | NA | NA | NA |  |
| **Brain metastasis** |  |  |  |  |  |  |  |  |  |  |
| [68Ga]Ga-Trivehexin | 12 | 0 | 1 | 0 | 92.3 (12/13) | NA | 100 (12/12) | NA | 92.3 (12/13) |  |
| [18F]FDG | 5 | 0 | 8 | 0 | 38.5 (5/13) | NA | 100 (5/5) | NA | 38.5 (5/13) |  |
| *p* value |  |  |  |  | **0.031** | NA | NA | NA | **0.031** |  |
| **Liver metastasis** |  |  |  |  |  |  |  |  |  |  |
| [68Ga]Ga-Trivehexin | 7 | 0 | 1 | 0 | 87.5 (7/8) | NA | 100 (7/7) | NA | 87.5 (7/8) |  |
| [18F]FDG | 8 | 0 | 0 | 0 | 100 (8/8) | NA | 100 (8/8) | NA | 100 (8/8) |  |
| *p* value |  |  |  |  | NA | NA | NA | NA | NA |  |
| **Other distant metastases**^a)^ | | | | | | | | | | |
| [68Ga]Ga-Trivehexin | 23 | 1 | 10 | 2 | 98.9 (23/33) | 66.7 (2/3) | 95.8 (23/24) | 16.7 (2/12) | 69.4 (25/36) |  |
| [18F]FDG | 28 | 2 | 5 | 1 | 84.9 (28/33) | 33.3 (1/3) | 93.3 (28/30) | 16.7 (1/6) | 80.6 (29/36) |  |
| *p* value |  |  |  |  | 1.000 | 1.000 | NA | NA | 0.774 |  |
| Note. a) Other distant metastatic locations include abdominal lymph node, lung, muscle, pancreas, peritoneum, and adrenal gland.  TP, true positive; FP, false positive; FN, false negative; TN, true negative; PPV, positive predictive value; NPV, negative predictive value; NA, not applicable.  **p* < 0.05 represents statistical difference. | | | | | | | | | | |

| **Supplementary Table S7 The changes of therapeutic management based on [68Ga]Ga-Trivehexin compared to [18F]FDG PET/CT in non-small cell lung cancer** | | | | | | | | | |
| --- | --- | --- | --- | --- | --- | --- | --- | --- | --- |
| **No. of**  **participants** | **Sex/Age** | **Pathological type** | **TNM-stage** | | | **[^68^Ga]Ga-Trivehexin *vs.* [^18^F]FDG** | **Therapeutic regimens** | | |
|  |  |  | **[^18^F]FDG** | **[^68^Ga]Ga-Trivehexin** | **Final TNM stage**^a)^ |  | **[^18^F]FDG** | **[^68^Ga]Ga-Trivehexin** | **Actual treatment** |
| 1 | M/46 | SCC | cT2bN1M0 (ⅡB) | cT2bN0M0 (ⅡA) | pT2bN0M0 (ⅡA) | Decrease | Surgery + adjuvant chemotherapy | Surgery | Surgery |
| 2 | M/64 | SCC | cT2bN3M0 (ⅢB) | cT2bN0M0 (ⅡA) | pT2bN0M0 (ⅡA) | Decrease | Radical concurrent chemoradiotherapy + immunotherapy | Surgery | Surgery |
| 3 | M/77 | SCC | cT3N3M0 (ⅢC) | cT3N0M0 (ⅡB) | pT3N0M0 (ⅡB) | Decrease | Radical concurrent chemoradiotherapy + immunotherapy | Surgery + adjuvant chemotherapy | Surgery + adjuvant chemotherapy |
| 4 | M/65 | SCC | cT2aN3M0 (ⅢB) | cT2aN0M0 (ⅠB) | pT2aN0M0 (ⅠB) | Decrease | Radical concurrent chemoradiotherapy + immunotherapy | Surgery | Surgery |
| 5 | M/60 | SCC | cT1cN2M0 (ⅢA) | cT1cN1M0 (ⅡB) | pT1cN0M0 (ⅠA3) | Decrease | Surgery + adjuvant chemotherapy + immunotherapy | Surgery + adjuvant chemotherapy | Surgery |
| 6 | M/51 | SCC | cT1cN2M0 (ⅢA) | cT1cN0M0 (ⅠA3) | pT1cN0M0 (ⅠA3) | Decrease | Surgery + adjuvant chemotherapy + immunotherapy | Surgery | Surgery |
| 7 | M/68 | SCC | cT2aN3M0 (ⅢB) | cT2aN0M0 (ⅠB) | pT2aN0M0 (ⅠB) | Decrease | Radical concurrent chemoradiotherapy + immunotherapy | Surgery | Surgery |
| 8 | M/64 | SCC | cT1cN3M0 (ⅢB) | cT1cN0M0 (ⅠA3) | pT1cN0M0 (ⅠA3) | Decrease | Radical concurrent chemoradiotherapy + immunotherapy | Surgery | Surgery |
| 9 | M/65 | ADC | cT2aN3M0 (ⅢB) | cT2aN1M0 (ⅡB) | pT2aN0M0 (ⅠB) | Decrease | Radical concurrent chemoradiotherapy + immunotherapy | Surgery + adjuvant chemotherapy | Surgery |
| 10 | F/68 | ADC | cT2aN3M0 (ⅢB) | cT2aN0M0 (ⅠB) | pT2aN0M0 (ⅠB) | Decrease | Radical concurrent chemoradiotherapy + immunotherapy | Surgery | Surgery |
| 11 | F/69 | ADC | cT1cN3M0 (ⅢB) | cT1cN0M0 (ⅠA3) | pT1cN0M0 (ⅠA3) | Decrease | Radical concurrent chemoradiotherapy + immunotherapy | Surgery | Surgery |
| 12 | M/65 | ASC | cT2aN3M0 (ⅢB) | cT2aN0M0 (ⅠB) | pT2aN0M0 (ⅠB) | Decrease | Radical concurrent chemoradiotherapy + immunotherapy | Surgery | Surgery |
| 13 | M/71 | SCC | cT1bN3M0 (ⅢB) | cT1bN0M0 (ⅠA2) | cT1bN0M0 (ⅠA2) | Decrease | Radical concurrent chemoradiotherapy + immunotherapy | Surgery | Stereotactic body radiation therapy |
| 14 | M/72 | SCC | cT3N2M0 (ⅢB) | cT3N0M0 (ⅡB) | cT3N0M0 (ⅡB) | Decrease | Surgery + adjuvant chemotherapy + immunotherapy | Surgery + adjuvant chemotherapy | Radical concurrent chemoradiotherapy |
| Note. ^a)^ Non-small cell lung cancer (NSCLC) was staged using the 8th AJCC TNM staging system. SCC, squamous cell carcinoma; ADC, adenocarcinoma; ASC, adenosquamous carcinoma; TNM, tumor-node-metastasis. | | | | | | | | | |

| **Supplementary Table S8 Assessment of correlation between tumor uptake on [^68^Ga]Ga-Trivehexin and [18F]FDG and immunohistochemistry in non-small cell lung cancer** | | | | | | | | | | | | | | |
| --- | --- | --- | --- | --- | --- | --- | --- | --- | --- | --- | --- | --- | --- | --- |
| **Parameters** | **Integrin β6** | | **TGFβ1** | | **Smad2** | | **Fibronectin** | | **Vimentin** | | **Ki-67** | | **MMP9** | |
|  | ***r*** | ***p* value** | ***r*** | ***p* value** | ***r*** | ***p* value** | ***r*** | ***p* value** | ***r*** | ***p* value** | ***r*** | ***p* value** | ***r*** | ***p* value** |
| **[^68^Ga]Ga-Trivehexin** | | | | | | | | | | | | | | |
| **SUVmax** | 0.631 | **< 0.001** | 0.587 | **0.001** | -0.554 | 0.096 | -0.121 | 0.739 | NA | NA | 0.351 | 0.320 | 0.080 | 0.825 |
| **SUVmean** | 0.579 | **0.001** | 0.593 | **< 0.001** | -0.622 | 0.055 | -0.043 | 0.906 | NA | NA | 0.240 | 0.504 | 0.020 | 0.956 |
| **ITV** | 0.248 | 0.178 | 0.237 | 0.200 | -0.449 | 0.193 | 0.493 | 0.148 | NA | NA | 0.739 | **0.015** | 0.121 | 0.740 |
| **TLI** | 0.460 | **0.009** | 0.414 | **0.020** | -0.509 | 0.133 | 0.320 | 0.367 | NA | NA | 0.652 | **0.041** | 0.080 | 0.825 |
| **TBR** | 0.528 | **0.002** | 0.551 | **0.001** | -0.502 | 0.139 | 0.147 | 0.685 | NA | NA | 0.511 | 0.131 | 0.101 | 0.782 |
| **TLR** | 0.646 | **< 0.001** | 0.553 | **0.001** | -0.622 | 0.055 | 0.112 | 0.757 | NA | NA | 0.400 | 0.252 | -0.101 | 0.782 |
| **[^18^F]FDG** | | | | | | | | | | | | | | |
| **SUVmax** | 0.326 | 0.157 | 0.438 | **0.014** | -0.262 | 0.464 | 0.528 | 0.117 | NA | NA | 0.757 | **0.011** | 0.442 | 0.201 |
| **SUVmean** | 0.274 | 0.136 | 0.449 | **0.011** | -0.262 | 0.464 | 0.528 | 0.117 | NA | NA | 0.702 | **0.024** | 0.382 | 0.276 |
| **MTV** | 0.285 | 0.121 | 0.284 | 0.121 | -0.509 | 0.133 | 0.320 | 0.367 | NA | NA | 0.665 | **0.036** | 0.141 | 0.698 |
| **TLG** | 0.373 | **0.039** | 0.416 | **0.020** | -0.390 | 0.266 | 0.510 | 0.132 | NA | NA | 0.708 | **0.022** | 0.161 | 0.657 |
| **TBR** | 0.148 | 0.427 | 0.329 | 0.071 | -0.390 | 0.266 | 0.510 | 0.132 | NA | NA | 0.702 | **0.024** | 0.281 | 0.431 |
| **TLR** | 0.214 | 0.249 | 0.331 | 0.069 | -0.390 | 0.266 | 0.510 | 0.132 | NA | NA | 0.739 | **0.015** | 0.281 | 0.431 |
| Note. SUV, standardized uptake value; ITV, integrin β6 expression tumor volume；TLI, total lesion integrin β6 expression; TBR, tumor-to-background (mediastinal blood pool) ratio; TLR, tumor-to-liver parenchyma ratio; MTV, metabolic tumor volume；TLG, total lesion glycolysis; NA, not available. | | | | | | | | | | | | | | |


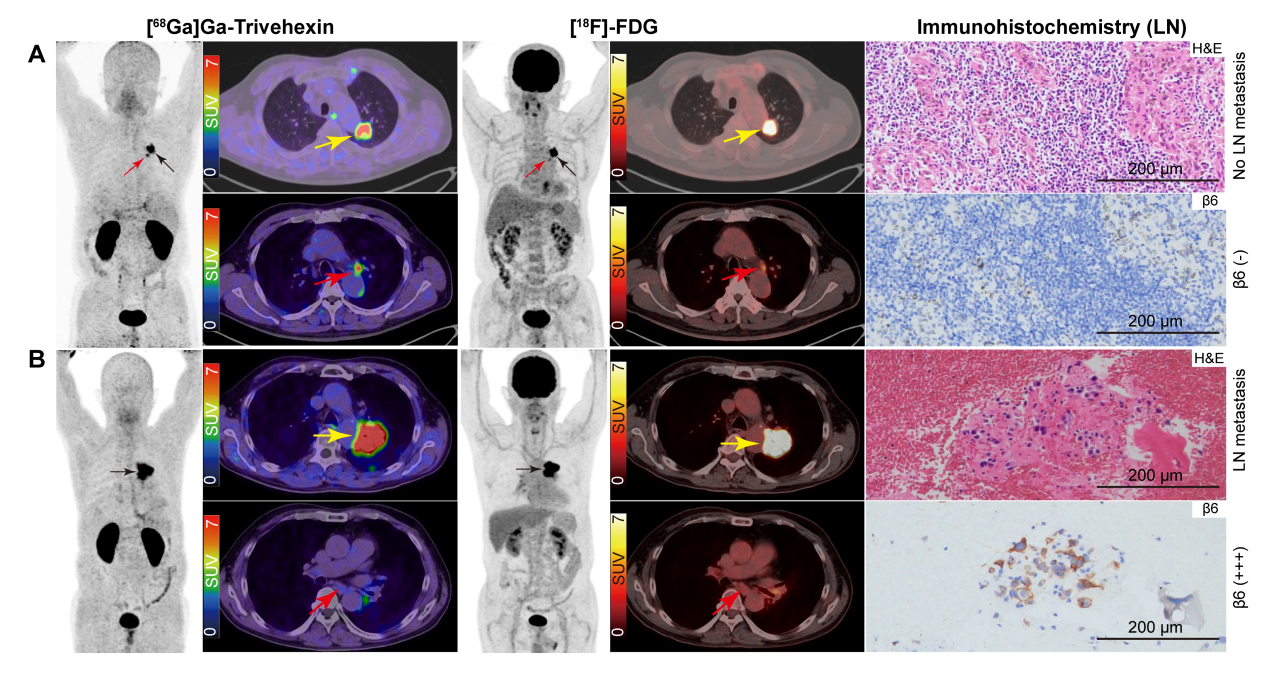
**Supplementary Figure S1.** Representative false-positive (A) and false-negative (B) lymph nodes (LN) on [68Ga]Ga-Trivehexin, hematoxylin-eosin (H&E), and integrin β6 immunostaining. (A) A 59-year-old man with left pulmonary sarcomatoid carcinoma (pT2aN1M0, ⅡB; black and yellow arrows). [68Ga]Ga-Trivehexin and [18F]FDG PET/CT showed the LN (station 5) with positive tracer uptake (SUVmax, 9.66 and 5.06, respectively; red arrows). Histopathological results confirmed the absence of metastasis to LN and negative integrin β6 expression. (B) A 60-year-old man with left lung adenocarcinoma (cT4N2M0, ⅢB; black and yellow arrows). Both [68Ga]Ga-Trivehexin and [18F]FDG PET/CT showed no tracer uptake in the mediastinal lymph node (station 7; red arrows). However, endobronchial ultrasound-guided transbronchial needle aspiration (EBUS-TBNA) biopsy confirmed metastasis to LN with strong integrin β6 expression. Magnification ×100; scale bar = 200 μm.

**
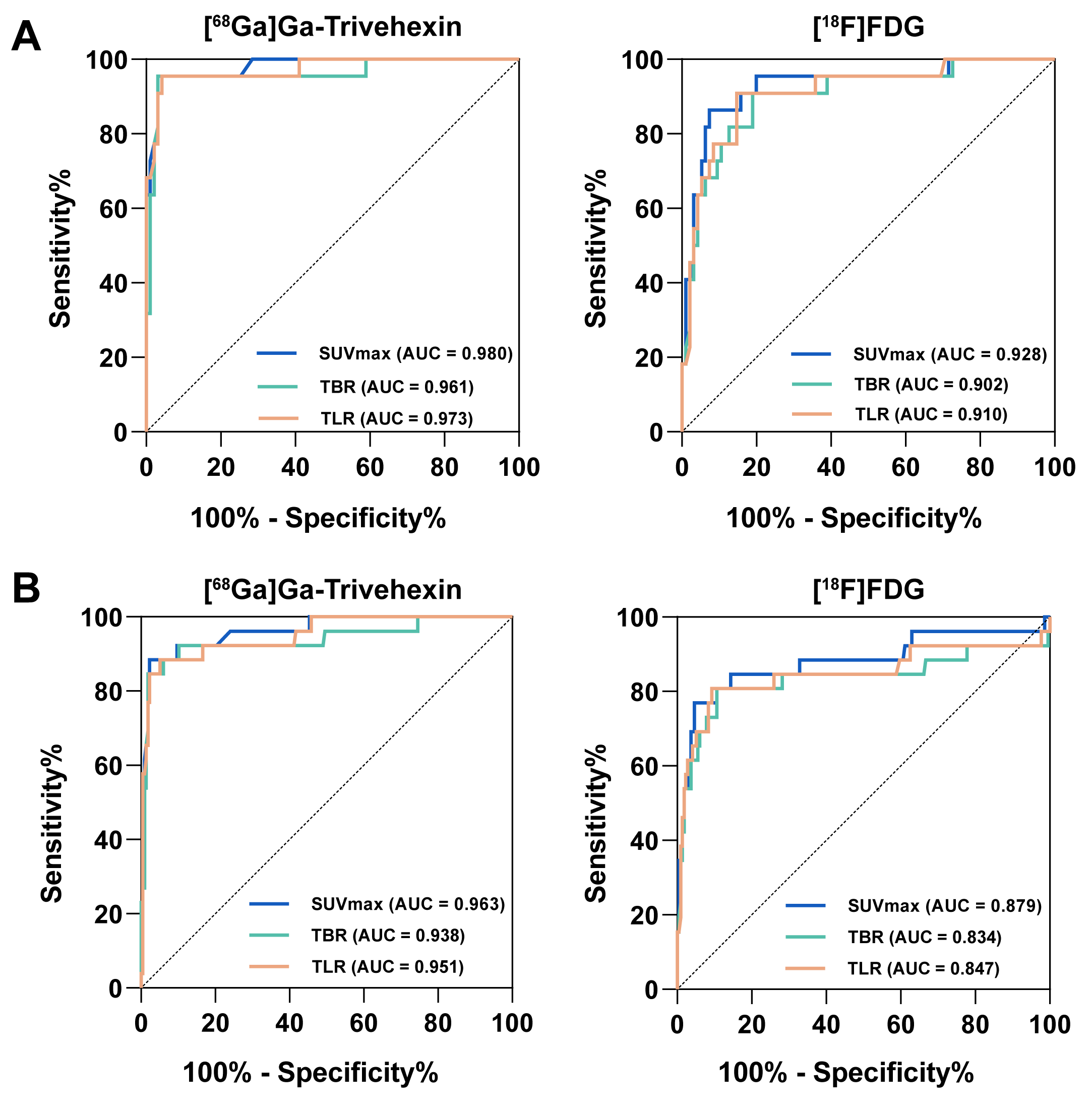
**

**Supplementary Figure S2.** Receiver operating characteristic (ROC) curves based on (A) station-based analysis and (B) lesion-based analysis for predicting lymph node metastasis on [^68^Ga]Ga-Trivehexin and [^18^F]FDG PET/CT in non-small cell lung cancer. AUC, area under the curve.


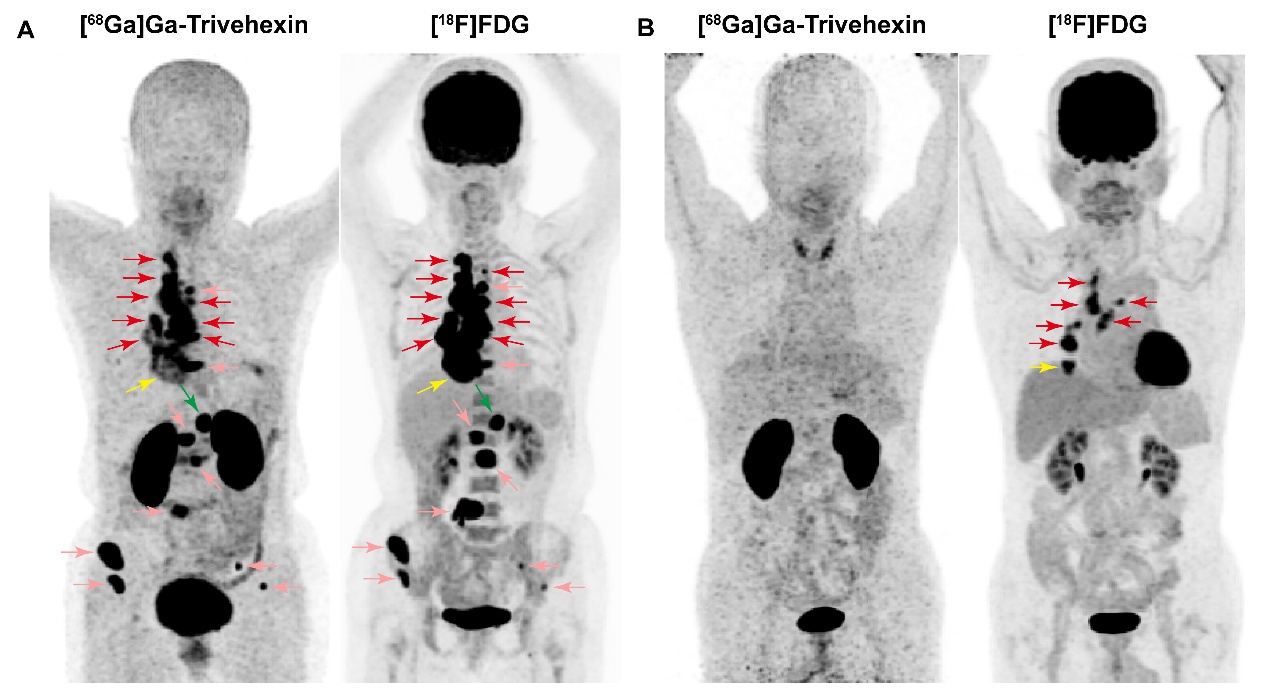


**Supplementary Figure S3.** Representative cases of non-small cell lung cancer (A) and small cell lung cancer (B). (A) A 61-year-old woman with right hilar non-small cell lung cancer (undefined type; cT4N3M1c, ⅣB). [^68^Ga]Ga-Trivehexin and [^18^F]FDG PET/CT showed the primary tumor (SUVmax, 11.21 vs. 47.22; yellow arrows) and metastatic lesions including lymph nodes (SUVmax, 4.02–11.21 vs. 40.74–50.56; red arrows), liver (SUVmax, 25.37 vs. 23.34; green arrows), and bone metastases (SUVmax, 6.17–30.88 vs. 6.16–39.90; pink arrows). (B) A 66-year-old man with small cell lung cancer in the right lower lobe (cT1N3M0, ⅢB). [^18^F]FDG PET/CT showed high tracer uptake in the primary tumor (SUVmax, 10.38; yellow arrow) and metastatic mediastinal lymph nodes (SUVmax, 6.29–9.27; red arrows). [^68^Ga]Ga-Trivehexin revealed false-negative findings.


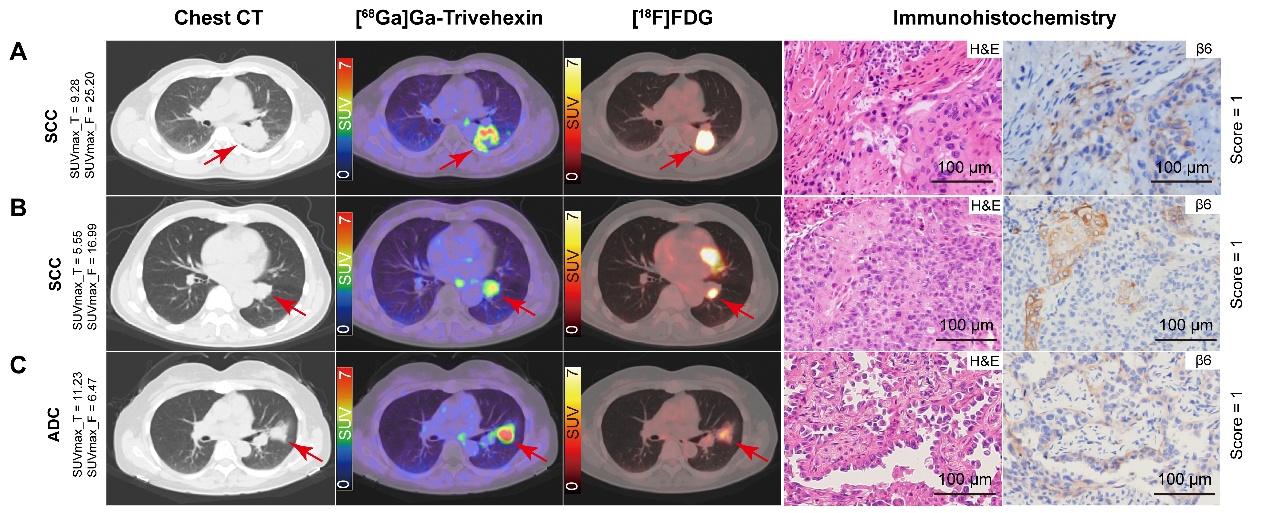


**Supplementary** **Figure S4.** Representative chest CT, [68Ga]Ga-Trivehexin, [18F]FDG PET/CT, hematoxylin-eosin (H&E), and integrin β6 immunostaining images of non-small cell lung cancer. (A) A 46-year-old man with squamous cell carcinoma (SCC; arrows) in the left lower lobe (pT2bN0M0, ⅡA). (B) A 60-year-old man with SCC (arrows) in the left upper lobe (pT1cN0M0, ⅠA3). (C) A 52-year-old woman with adenocarcinoma (ADC; arrows) in the left upper lobe (pT2aN2M0, ⅢA). Magnification × 100; scale bar = 100 μm. SUVmax_T, [68Ga]Ga-Trivehexin-derived SUVmax; SUVmax_F, [18F]FDG-derived SUVmax.


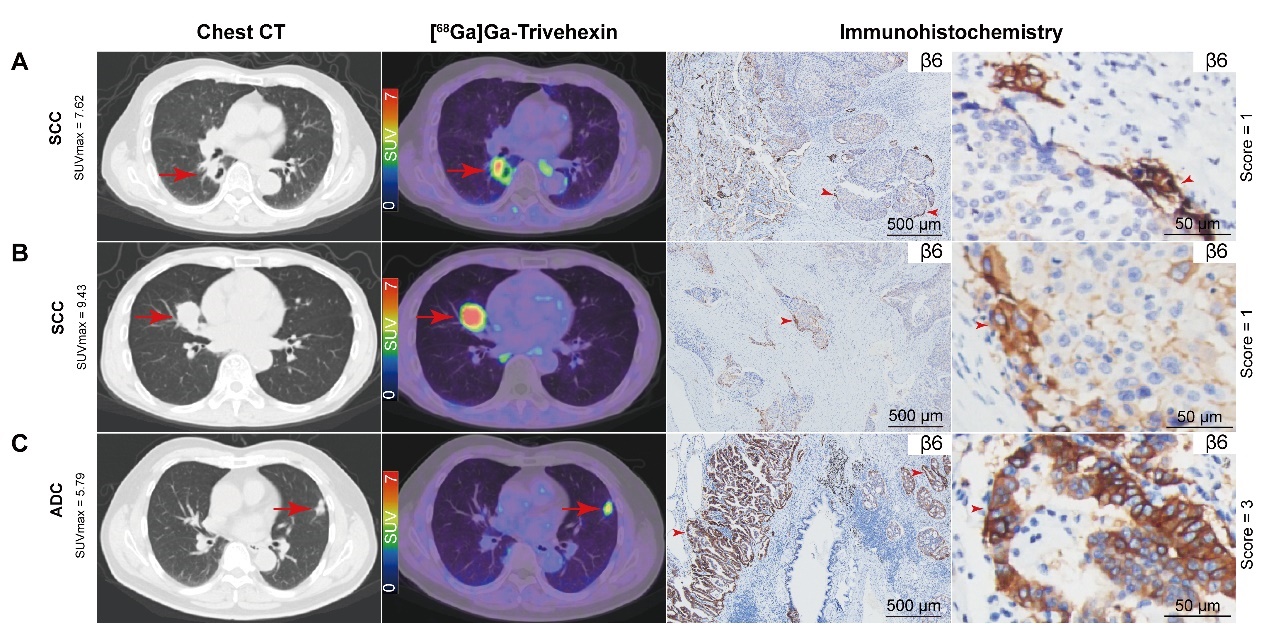
**Supplementary** **Figure S5** Representative chest CT, [68Ga]Ga-Trivehexin PET/CT and integrin β6 immunostaining images. (A) A [68Ga]Ga-Trivehexin-avid squamous cell carcinoma (SCC; pT2aN0M0, ⅠB; arrows) in the right lower lobe in a 65-year-old man showed weak integrin β6 expression. (B) A [68Ga]Ga-Trivehexin-avid SCC (pT2bN0M0, ⅡA; arrows) in the right middle lobe in a 60-year-old man revealed mild integrin β6 expression. (C) A [68Ga]Ga-Trivehexin-avid adenocarcinoma (ADC; pT2aN0M0, ⅠB; arrows) in the left upper lobe in a 65-year-old man exhibited intense integrin β6 expression. (A–C) Immunohistochemistry demonstrated that the tumor cells close to the tumor stroma showed strong integrin β6 expression, whereas the inner tumor cells showed weak or relatively weak expression (small red arrows). Scale bar = 500 μm and 50 μm, respectively.


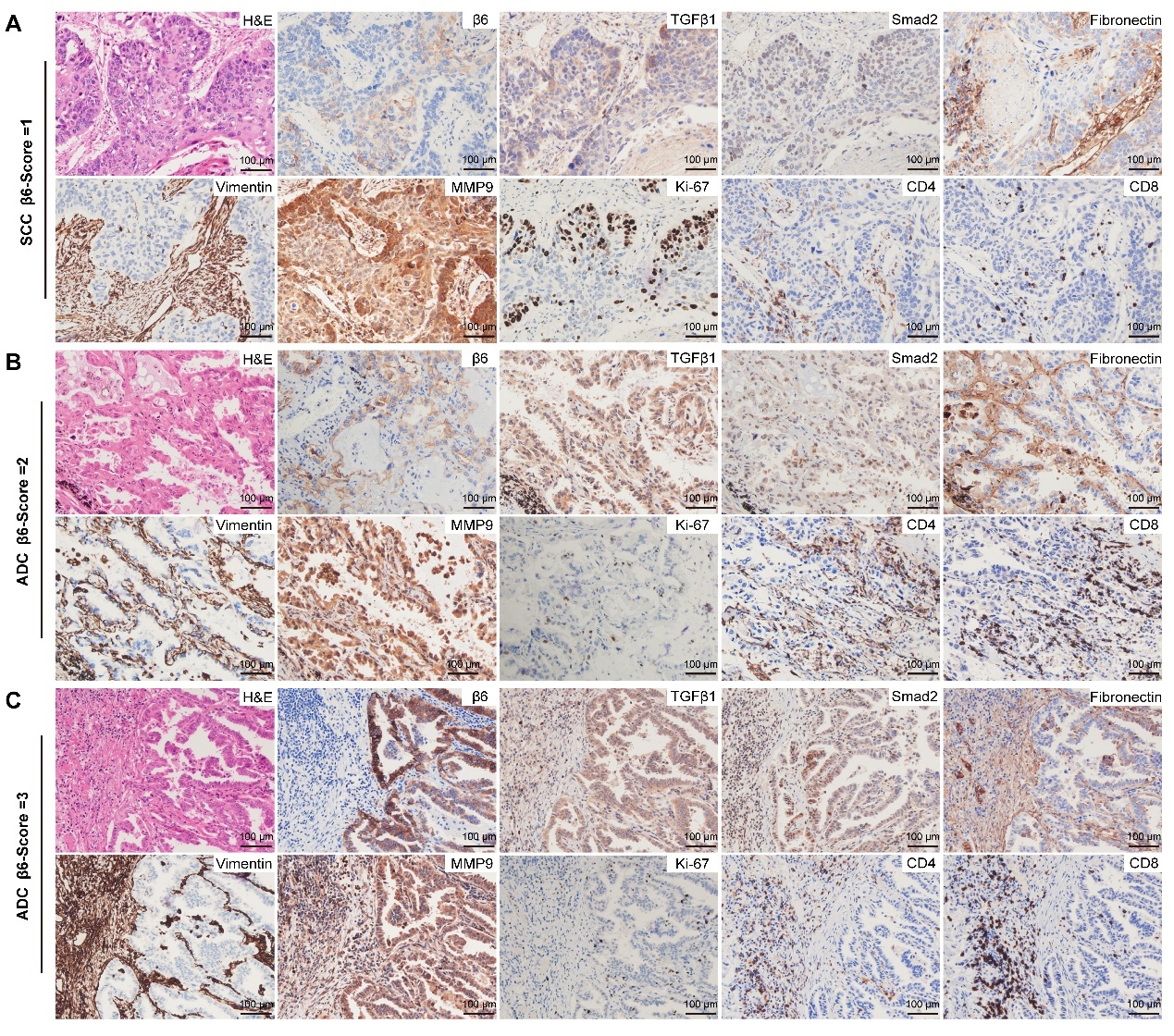


**Supplementary Figure S6** Representative hematoxylin-eosin (H&E) and immunohistochemical staining of integrin β6, TGFβ1, Smad2, fibronectin, vimentin, MMP9, Ki-67, CD4, and CD8 in (A) lung squamous cell carcinoma (SCC) and (B–C) adenocarcinoma (ADC). Magnification ×100; scale bar = 100 μm.


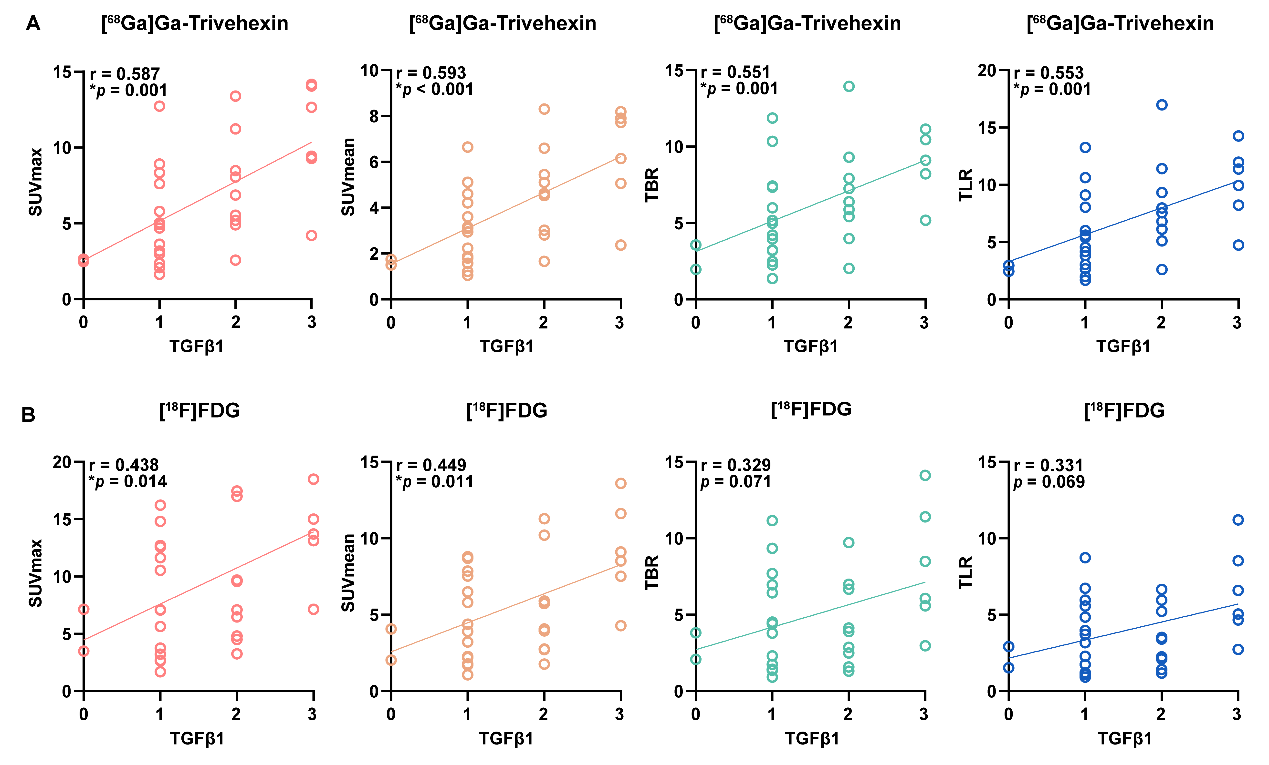


**Supplementary Figure S7** Assessment of the correlation between tumor tracer uptake on [68Ga]Ga-Trivehexin (A) and [18F]FDG (B) and tissue TGFβ1 expression in non-small cell lung cancer. *P*-values were determined via Spearman correlation coefficient (**p* < 0.05). TBR, tumor-to-background (mediastinal blood pool) ratio; TLR, tumor-to-liver parenchyma ratio.


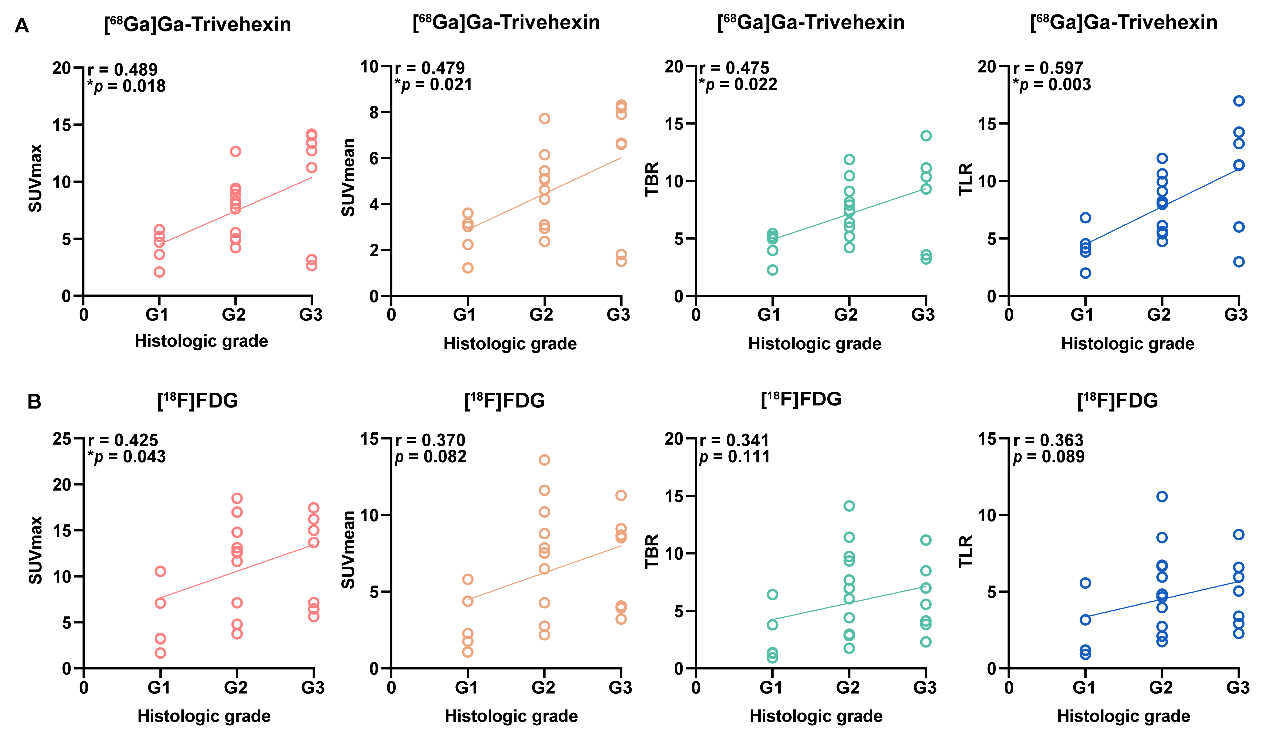


**Supplementary Figure S8** Assessment of the correlation between tumor tracer uptake on [68Ga]Ga-Trivehexin (A) and [18F]FDG (B) and tumor histologic grade in non-small cell lung cancer. *P*-values were determined via Spearman correlation coefficient (**p* < 0.05). TBR, tumor-to-background (mediastinal blood pool) ratio; TLR, tumor-to-liver parenchyma ratio.


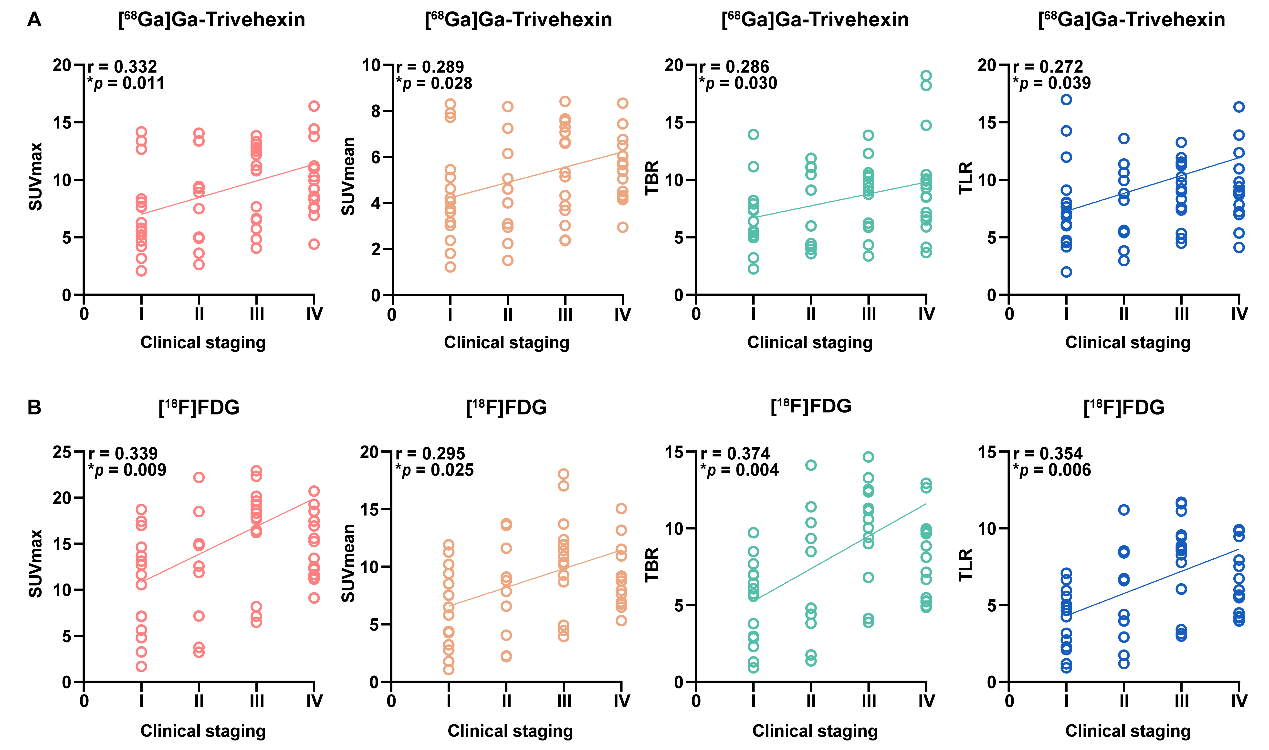


**Supplementary Figure S9** Assessment of the correlation between tumor tracer uptake on [68Ga]Ga-Trivehexin (A) and [18F]FDG (B) and clinical staging in non-small cell lung cancer. *P*-values were determined via Spearman correlation coefficient (**p* < 0.05). TBR, tumor-to-background (mediastinal blood pool) ratio; TLR, tumor-to-liver parenchyma ratio.
